# A new classification of cardio-oncology syndromes

**DOI:** 10.1186/s40959-021-00110-1

**Published:** 2021-06-21

**Authors:** Rudolf A. de Boer, Joseph Pierre Aboumsallem, Valentina Bracun, Douglas Leedy, Richard Cheng, Sahishnu Patel, David Rayan, Svetlana Zaharova, Jennifer Rymer, Jennifer M. Kwan, Joshua Levenson, Claudio Ronco, Paaladinesh Thavendiranathan, Sherry-Ann Brown

**Affiliations:** 1 Department of Cardiology, University Medical Center Groningen, University of Groningen, Groningen, the Netherlands; 2 Division of Cardiology, Department of Medicine, University of Washington, Seattle, WA USA; 3 Department of Medicine, Medical College of Wisconsin, Milwaukee, WI USA; 4 Cardio-Oncology Program, Division of Cardiovascular Medicine, Medical College of Wisconsin, 8701 Watertown Plank Road, Milwaukee, WI 53226 USA; 5 Duke University, Durham, NC USA; 6 Section of Cardiovascular Medicine, Yale School of Medicine, New Haven, CT USA; 7 Heart and Vascular Institute, University of Pittsburgh Medical Center, Pittsburgh, PA USA; 8 Department of Medicine, University of Padova, Padova, Italy; 9 International Renal Research Institute of Vicenza, Vicenza, Italy; 10 Department of Nephrology, San Bortolo Hospital, Vicenza, Italy; 11 Division of Cardiology, Toronto General Hospital, Toronto, Canada

**Keywords:** Cardio-oncology, Cancer, Cardiac Tumours, Prevention, Cardiotoxicity, Inflammation, Cardiovascular disease, Immune system, Heart failure, Prognostic biomarkers, Medical diagnostic radiation

## Abstract

Increasing evidence suggests a multifaceted relationship exists between cancer and cardiovascular disease (CVD). Here, we introduce a 5-tier classification system to categorize cardio-oncology syndromes (COS) that represent the aspects of the relationship between cancer and CVD. COS Type I is characterized by mechanisms whereby the abrupt onset or progression of cancer can lead to cardiovascular dysfunction. COS Type II includes the mechanisms by which cancer therapies can result in acute or chronic CVD. COS Type III is characterized by the pro-oncogenic environment created by the release of cardiokines and high oxidative stress in patients with cardiovascular dysfunction. COS Type IV is comprised of CVD therapies and diagnostic procedures which have been associated with promoting or unmasking cancer. COS Type V is characterized by factors causing systemic and genetic predisposition to both CVD and cancer. The development of this framework may allow for an increased facilitation of cancer care while optimizing cardiovascular health through focused treatment targeting the COS type.

## Introduction

With 2 million new cancer diagnoses per year, improvements in cancer therapies has led to an increasing prevalence of cancer survivorship [1]. There are close to 17 million cancer survivors in the United States today, representing 1 in 20 people [1, 2]. In the next 10 years, there is a projected 31% increase in survivors, with more than 22 million cancer survivors estimated for 2030 [1]. In the last decade, cardiologists and oncologists have provided clinical and experimental evidence that cancer, as well as cancer treatments, result in detrimental effects on the cardiovascular system, a consequence that imposes clinical challenges for patient management. In parallel, the captivating notion that CVD can represent a pre-oncogenic condition has gained growing awareness [3]. Among survivors, CVD is the leading cause of non-cancer related mortality [4, 5]. Interestingly, the relationship is not unidirectional [6]. Epidemiologic data have reported an increased cancer risk in patients with CVD [7–9]. This relationship may be intuitive, given that both disease processes share common risk factors and pathogenesis.

**Fig. 1:**
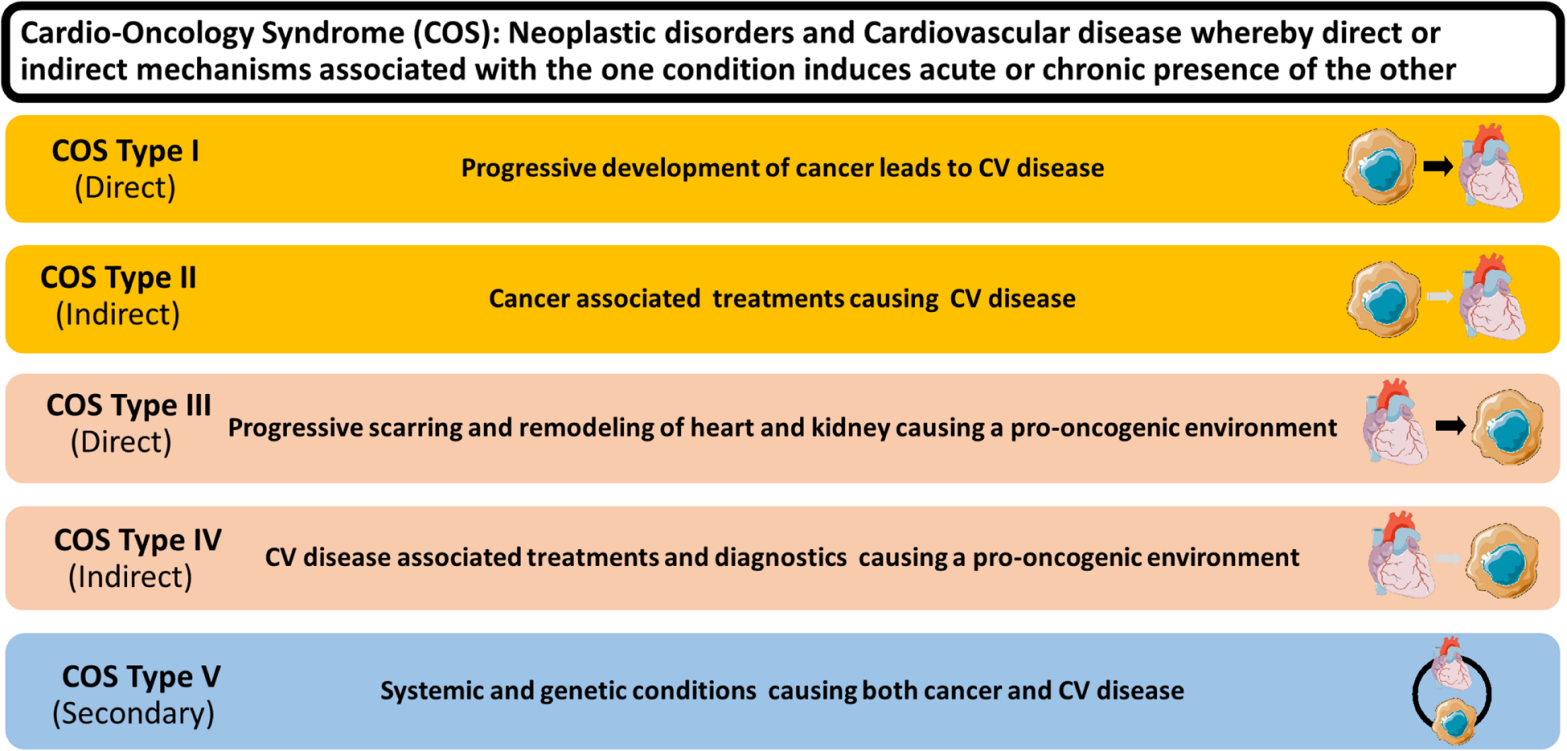
Cardio-Oncology Syndromes in Precision Cardio-Oncology. The five types of Cardio-Oncology Syndromes are listed, along with a description of whether they are direct (black arrows), indirect (grey arrows), or secondary and how they are defined. A few examples are given for each type. CHiP, clonal haematopoiesis of indeterminate potential; COS, Cardio-Oncology Syndromes; CV, cardiovascular; TKI, Tyrosine kinase inhibitors; VEGF, vascular endothelial growth factor

**Fig. 2:**
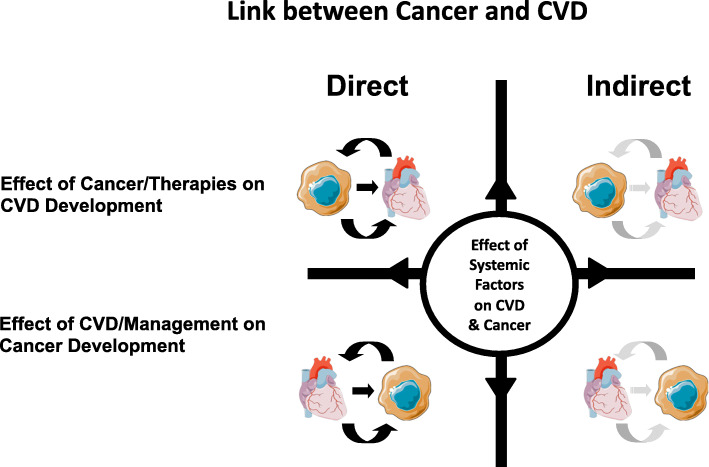
Relationships between CVD and cancer. This schematic demonstrates the multifaceted relationship that exists between CVD and cancer. While CVD is the leading cause of non-cancer related mortality among cancer survivors, there is also a known increased risk for cancer in patients with CVD. Additionally, CVD and therapies for CVD can have both a direct (curved black arrows) and indirect (curved grey arrows) impact on cancer; while cancer and therapies for cancer can have both a direct (curved black arrows) and indirect (curved grey arrow) impact on cardiac function and health. CVD, cardiovascular disease

In this manuscript, we propose a 5-tier classification system to better characterize the intersection between cardiology and oncology (Fig. 1). Types I and II of our classification systems address how cancer and cancer therapies affect the cardiovascular system, while Types III and IV incorporate how cardiovascular diseases, monitoring strategies, and therapies may contribute to unmasking cancer or fostering a tumorigenic environment (Fig. 2). Type V addresses systemic and genetic conditions that can lead to both CVD and cancer. We also emphasize the need for medical communities in both cardiology and oncology to raise awareness of these aetiologies and related challenges to maximize collaboration among medical scientists and clinicians. Such interdisciplinary partnerships will help advance the clinical care, research, and education needed in Cardio-Oncology to advance the field and optimize the care landscape for our patients.

## Cardio-oncology syndrome type I (COS 1; direct): effect of the presence of cancer itself on the cardiovascular system

### Arterial thromboembolism due to cancer

**Fig. 3:**
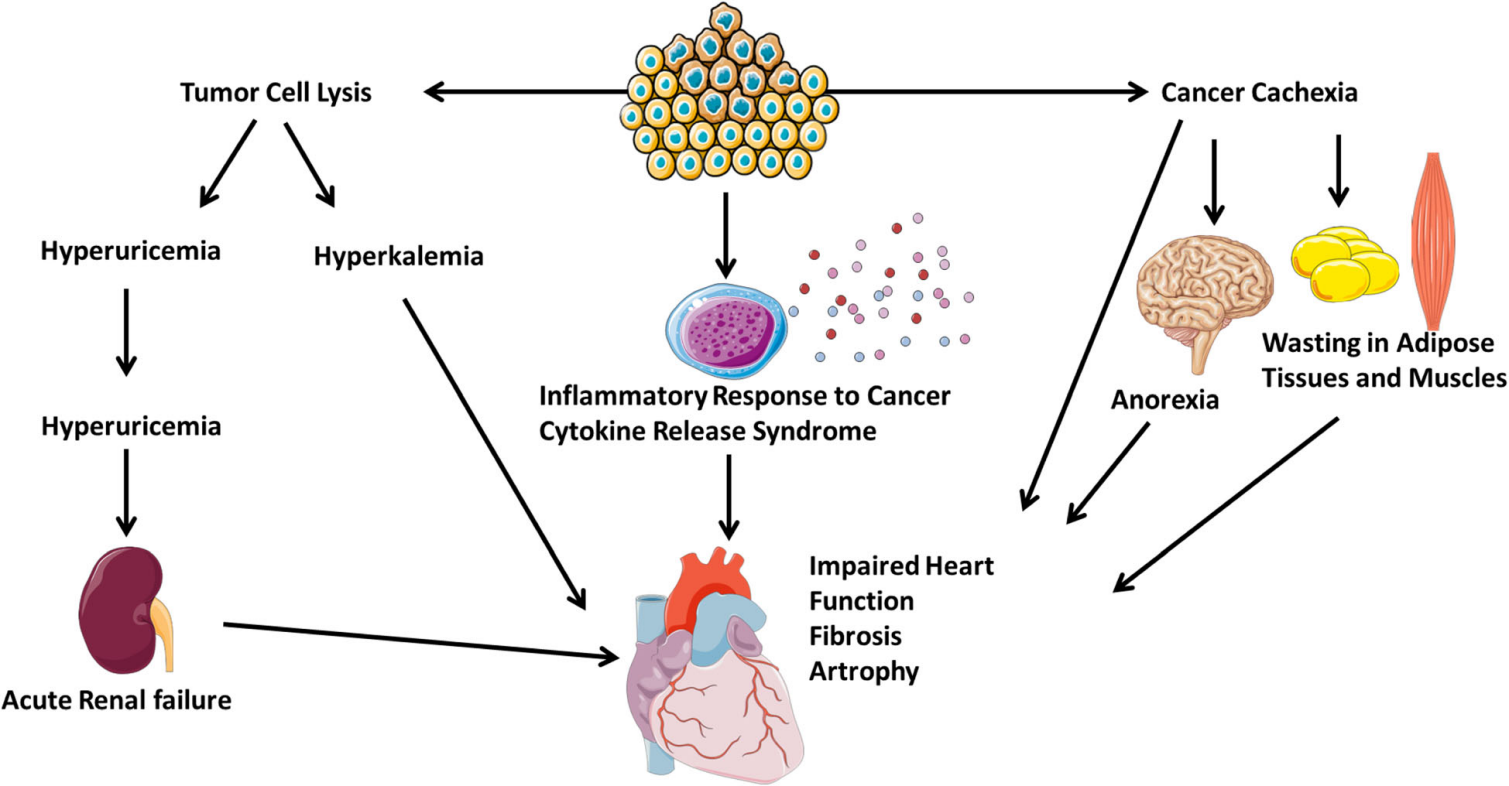
Mechanisms by which the development of cancer can lead to CVD (Cardio-Oncology Syndrome Type I). There are three primary mechanisms demonstrated in this schematic to illustrate COS Type I. Patients with cancer have a higher risk for both venous and arterial thromboembolism. Patients with sudden development of cancer may develop tumour lysis syndrome, whereby hyperuricemia may result in acute kidney injury which may have a deleterious effect on cardiac function, and severe hyperkalaemia may result in arrhythmias. Finally, the inflammatory mediators from cancer cachexia may have a negative impact on cardiac function. CVD, cardiovascular disease

Patients with cancer are at higher risk of developing venous and arterial thromboembolic events. The risk of arterial thromboembolism varies by cancer type [10, 11]. Particularly lung, gastric, and pancreatic cancers demonstrate the higher risk. Evidence for this statement is substantial, while the mechanism remains less clear. It has been suggested that tumour cells present tissue factors and are therefore able to bind with coagulation factors. Concurrently, they also produce inflammatory cytokines and cancer procoagulant factors [12]. These mechanisms are responsible for the activation of the coagulation-cascade, promotion of the platelet activation and consequentially increased thrombus formation. Furthermore, patients with cancer often present with lymphocytosis, neutrophilia, and thrombocytosis, which are known to promote hypercoagulability (Fig. 3) [13].

### Cancer-associated cachexia and anorexia

Cachexia is a state of involuntary weight loss and is commonly observed in patients with cancer - predominantly pancreatic, colon, lung, head, and neck as well as in gastro-oesophageal cancer. Depending on the cancer type, the prevalence of cachexia varies between 40 to 70%. Though this condition impacts several organ systems, skeletal muscle is the first to be affected by body wasting (Fig. 3) [14]. The cardiac manifestations, as shown primarily in animal studies, results in altered heart function, cardiac atrophy, and fibrosis, and reduced cardiac weight [15].

The mechanisms by which malignancies induce cardiac wasting are not fully elucidated. It has been proposed the ubiquitin-proteasome system, autophagy, and myocyte apoptosis contribute to cardiac wasting [16]. Also, cancer-associated cachexia is characterized by the secretion of inflammatory mediators and hormonal factors from tumours and their microenvironment [15]. These factors demonstrate diagnostic value and may serve as biomarkers for the identification of cancer-induced cardiac alterations.

Anorexia is a major component of cancer cachexia. Tumours cause dysphagia and alter the gut function resulting in nutritional deficiencies. In addition, some cancer patients experience depression and pain, further decreasing the desire to eat [17]. Eating disorders have multiple medical consequences, such as potentially life-threatening cardiovascular complications characterized by hemodynamic and structural changes, cardiomyopathy, and premature death [18].

### Tumour lysis syndrome

Tumour lysis syndrome (TLS) describes a state of a massive tumour cell death resulting in the development of metabolic imbalances and organ dysfunction. TLS can occur spontaneously when cancer cells die without preceding chemotherapy, embolization, or radiation therapies, or as a result of antineoplastic treatments. The majority of TLS cases are reported in haematological malignancies, such as leukaemia and lymphomas with marked sensitivity to cell lysis with chemotherapy. The prevalence of TLS is lower in patients with solid cancer. The pathogenesis of TLS is explained by the fact that cells, particularly malignant cells, contain high levels of potassium, phosphorus, and uric acid. The release of these intracellular substances facilitates the development and progression of TLS and its complications [19].

Cancer cell lysis is associated with a significant release of nucleic acids, purines, and eventually uric acid. The latter can crystalize and block the flow in the renal tubules, which consequently leads to acute kidney injury (Fig. 3) [20]. Hyperuricemia is associated with impaired endothelium-mediated relaxation, vascular stiffness and hampering left ventricular filling pattern. Additionally, serum levels of uric acid are linked to the progression of heart failure (HF). Excessive phosphorus binds to calcium and forms a calcium-phosphorus product called calcium phosphate. This product is deposited in kidneys and cardiac tissue resulting in acute kidney injury and cardiac arrhythmia, respectively. Also, the decline in free calcium concentration, caused by phosphorus binding, is associated with prolongation of the QT interval on ECG and muscle tetany. This may lead to arrhythmias if drugs that prolongate the QT interval are administrated [20].

## Cardio-oncology syndrome type II-(COS 2; indirect): anti-neoplastic treatments cause acute or chronic CV disease

### Cancer treatments-induced cardiotoxicity

**Fig. 4:**
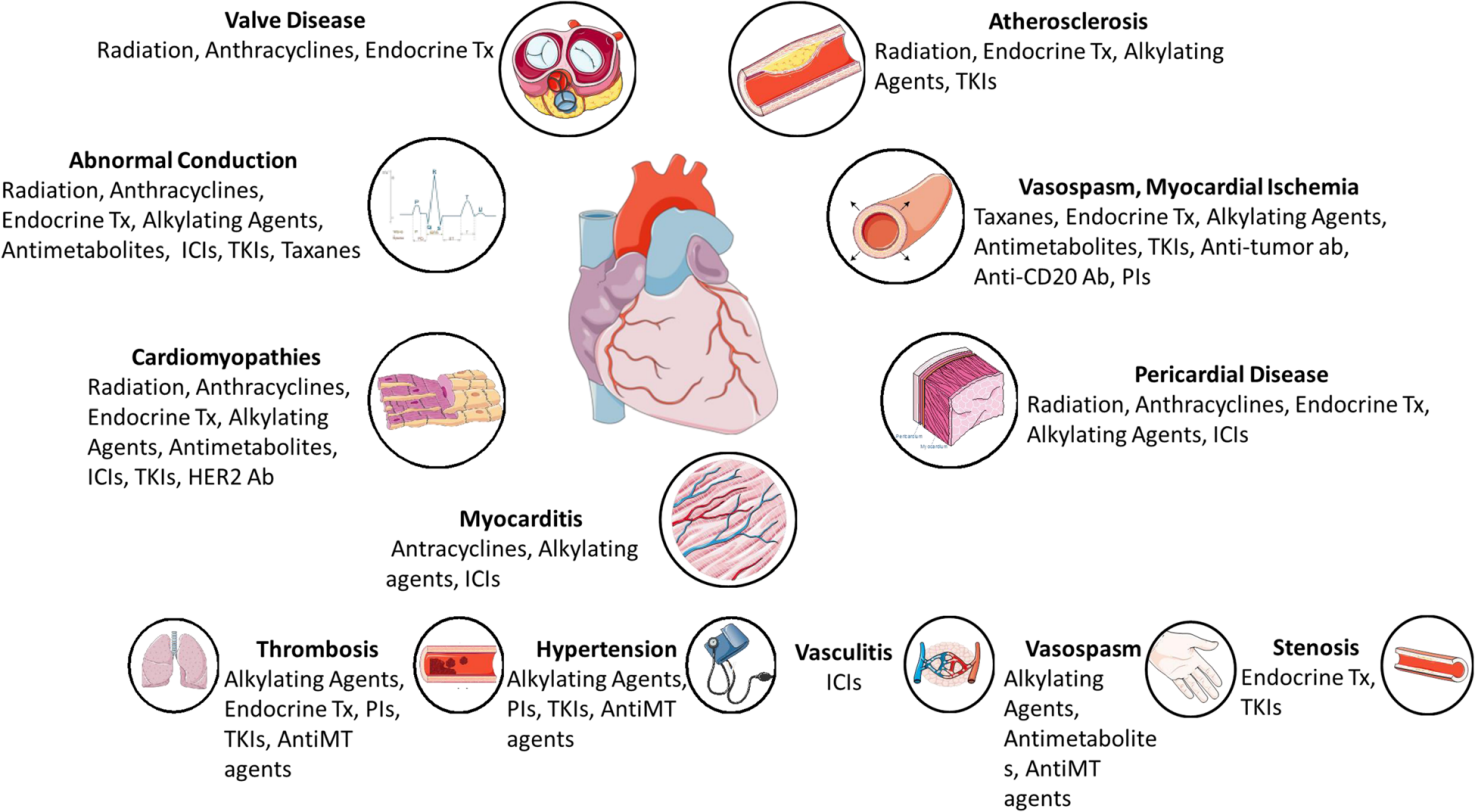
Cardiovascular toxicities of cancer therapies. Cardiovascular toxicities of a wide spectrum of cancer therapies can affect the heart (center, left, and right of figure) or peripheral vasculature (bottom row of figure). Ab, antibody; CDK, cyclin dependent kinase; HER2, human epidermal growth factor receptor 2; ICI, immune checkpoint inhibitor; MT, microtubule; PI, platinum inhibitor; TKI, tyrosine kinase inhibitor; Tx, Therapy. Used with permission; from Brown [23], Creative Commons Attribution License [CC BY]

**Fig. 5:**
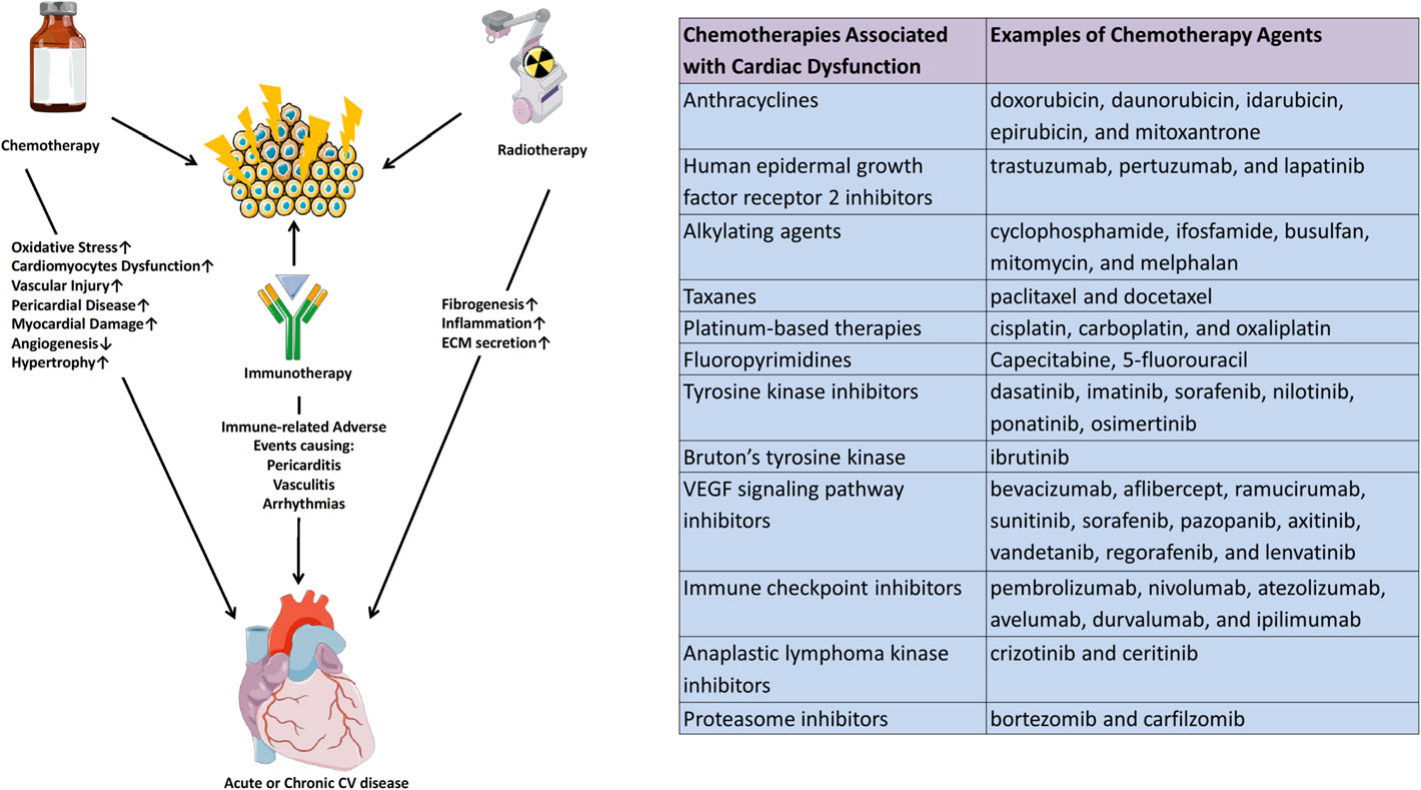
Mechanisms by which anti-neoplastic therapies may result in CVD (Cardio-Oncology Syndrome Type II). There are many examples shown here in which anti-neoplastic therapies may result in acute or chronic CVD [22]. Pharmacotherapies, such as anthracyclines and HER2 receptor antagonists may result in myocardial dysfunction and heart failure. Immunotherapy can also induce cardiotoxicity. Radiation therapy may also result in the development of atherosclerosis, as well as damage to the heart valves or pericardium. CV, cardiovascular; ECM, extracellular matrix; HER2, human epidermal growth factor receptor 2. Adapted with permission; from Brown [23], Creative Commons Attribution License [CC BY]

Modern cancer therapies can lead to CV structural and functional dysfunction [21]. Cardiotoxicity is one of the most concerning side effects of chemotherapy. The classification and segregation of toxicities by type of therapy have been discussed extensively elsewhere [22] and are largely catalogued in Fig. 4. This section highlights a few examples of cancer therapy-related cardiac dysfunction. A broad list of commonly used chemotherapies is provided in Fig. 5.

Several classes of commonly used chemotherapy agents are known to have myocardial dysfunction and HF as important consequences, most notoriously anthracyclines and human epidermal growth factor receptor 2 (HER2) receptor antagonists [24]. While both agents can cause reversible myocardial dysfunction, irreversible myocyte damage is classically reported with anthracyclines and can lead to HF years after drug administration. Even “targeted” therapy such as vascular endothelial growth factor inhibitors can exert off-target effects causing hypertension, thromboembolism, QT prolongation, and atrial fibrillation (AF), as well as cardiomyopathy [24–27]. Other targeted tyrosine kinase inhibitors such as ibrutinib, sorafenib, and mammalian target of rapamycin complex (mTORC) inhibitors also have a wide range of CV effects including AF, QTc prolongation, and HF [28]. Cancer patients can face an increase in venous thromboembolism compared to the general public, depending on the type of cancer or specific treatments, among other factors [10]. The pharmacokinetics of several chemotherapeutic agents, coalescing with drug interactions, frequent surgical procedures, and reduced mobility of patients during treatment also contribute to the risk for thromboembolism.

Different conditions can be induced by immunotherapies for onco-hematological disorders. As an example, chimeric antigen receptor T (CAR-T) cell therapy is used in the treatment of haematological malignancies, including acute leukaemia, lymphoma and multiple myeloma. In spite of the remarkable clinical effectiveness, this treatment shows serious side effects that cannot be underrated. Cytokine release syndrome (CRS) is one of the most clinically important and potentially harmful toxicities. When CAR-T cells are activated by malignant cell antigen, they in turn activate monocytes and macrophages, causing the release of proinflammatory cytokines and chemokines including IL-6, IL-8, IL-10, interferon-gamma (INF-y), monocyte chemoattractant protein-1b, and granulocyte-macrophage colony-stimulating factor [29, 30]. Consequently, the IL-6 receptor antagonist Tocilizumab is used to treat CRS in cancer patients as first-line treatment [31–34]. While cardiac dysfunction caused by CRS is largely reversible, tocilizumab is used in more severe cases, which can include lethal cardiac dysfunction [35, 36]. Consequently, tocilizumab, an IL-6 receptor antagonist, is indicated when patients demonstrate significant left ventricular dysfunction [37]. Corticosteroid therapy is applied in certain acute cases that do not respond to tocilizumab. When a patient may not respond to tocilizumab or a steroid, other agents such as anakinra (an IL-1R inhibitor) and etanercept (an anti-TNF) can be used to block inflammatory pathways [29, 38]. Indeed, immunotherapy can trigger native immune-system mediated cardiotoxicity, such as myocarditis, pericarditis, HF, and arrhythmias [10]. In general, these events are uncommon, occurring in < 3% of patients who receive immune checkpoint inhibitors, but carry a high risk of morbidity and mortality [39].

Radiation therapy is also taxing on the CV system. Radiotherapy may lead to both short and long-term epicardial coronary artery and microcirculatory damage, may cause regurgitation related to valve retraction and stenosis, and can induce fibrotic changes in the parietal pericardium [24].

## Cardio-oncology syndrome type III (COS 3; direct): CVD promotes a pro-oncogenic environment

### Epidemiological evidence demonstrating higher cancer risk in CVD patients

Emerging epidemiological data has suggested an increased cancer risk in patients with prevalent CVD compared to subjects without CVD [3, 7–9]. Even though most studies attempt to account for surveillance bias, this remains a potential concern with many observational studies. In a Danish study, all age groups demonstrated higher incidence rates of cancer after 1 year from the diagnosis of MI [40]. The association of CVD with increased cancer risk is further supported by a long-term prospective study that evaluated the clinical features and prevalence of malignant neoplasia in patients with acute coronary syndrome (ACS) during a 17-year follow-up. The incidence rate was 17.8 cases per 1000 person-years in patients with ACS, which was three times higher than that observed in the general population. Those who developed malignancies after the ACS diagnosis demonstrated a worse prognosis [41].

Earlier, data of the Swedish Inpatient Register were collected from patients who were admitted to the hospital between the years 1965 and 1983 for venous thromboembolism. The enrolled patients did not have cancer at the start of the study. The authors stated that venous thromboembolism could be a potential indicator of cancer as 4% of these patients were diagnosed with cancer within the first year after admission and the risk of all cancers was higher than expected with a standardised incidence rate of 4.4 (95% CI 4.2–4.9) [42].. Based on data collected for the Vitamin Intervention for Stroke Prevention study, the investigators revealed that ischemic stroke survivors had a higher annual rate of age-adjusted cancer risk compared to the general population at 1 year (581.8/100,000 persons vs. 486.5/100,000 persons, SIR 1.2, 95% CI 1.16–1.24) and 2 years (1301.7/100,000 vs. 911.5/100,000, SIR 1.4, 95% CI 1.2–1.6) after recruitment [43]. In these instances, the temporal presentation of events helps to define the sequence of risk.

Based on 13 prospective studies, a meta-analysis found patients with hypertension were more susceptible to develop breast cancer [44, 45]. The prevalence of colorectal cancer was 11% higher in patients with hypertension and renal cell carcinoma was also shown to be associated with high blood pressure [44].

Interestingly, AF may be also associated with cancer. The RE-LY (Randomized Evaluation Long-Term Anticoagulant Therapy Study) revealed that malignant tumours were the main non-CV cause of mortality in AF patients [46, 47]. Also, the Women’s Health Study (WHS) showed that 10% of patients who had new-onset AF developed subsequent cancer [47]. Yet, most evidence stems from retrospective analyses with mostly non-causal relationships. Also, available data tend to show positive associations due to publication bias. Thus, these findings should be examined and evaluated critically by the reader.

An additional consideration is potential survivor bias in cardiovascular trials. Participants that receive and survive focused cardiac treatment or intervention will accrue exposure-time for risk of developing subsequent cancer. However, since most studies compared those with CVD compared to those without CVD when assessing the association of CVD with subsequent risk for cancer, it is unlikely the association seen between CVD and cancer is driven by survivor bias, since those with CVD even with treatment, are less likely to accrue more time at risk than the non-CVD comparator.

### The effect of CVD-induced hypoxia on cancer

**Fig. 6:**
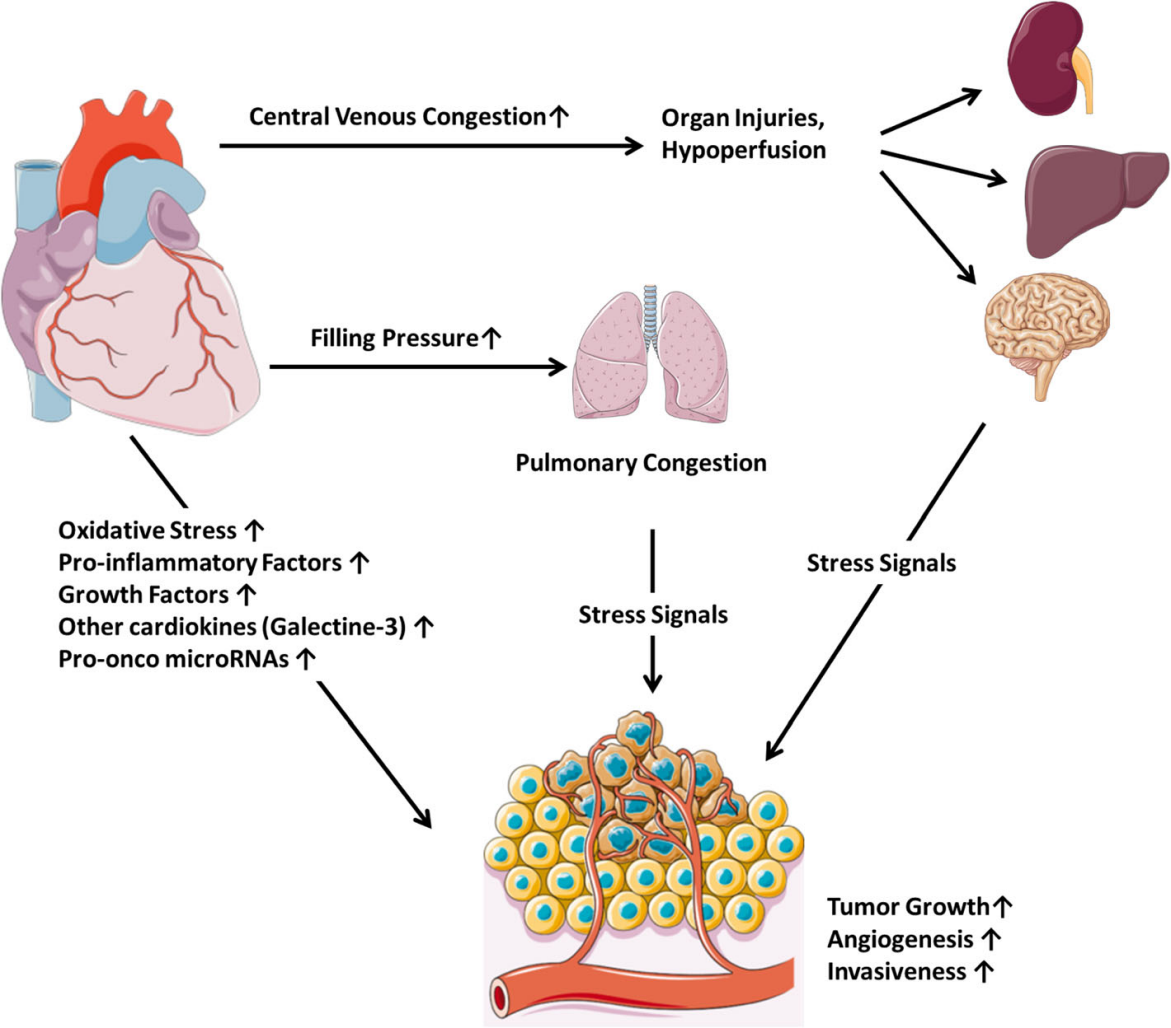
Mechanisms by which cardiac scarring and remodelling may promote a pro-oncogenic environment (Cardio-Oncology Syndrome Type III). This schematic demonstrates how a pro-oncogenic environment can result from cardiac dysfunction. With cardiac dysfunction (i.e. development of heart failure after a myocardial infarction), increased central venous congestion can result in hypoperfusion for various end organs, which then release stress signals that may increase tumour growth, angiogenesis, and tumour invasiveness. Increased filling pressures may similarly result in pulmonary congestion and the increase in stress signals. Cardiac dysfunction may also result in release of cardiokines, increased oxidative stress, and pro-inflammatory factors which may result in stimulated tumour growth, angiogenesis, and tumour invasiveness

Evidence suggests that tissue hypoxia in atherosclerosis may accelerate cancer progression (Fig. 6). Endothelial abnormalities and insufficient blood flow induce tissue hypoxemia in CVD patients. Hypoxia subsequently induces hypoxia-inducible factor 1-alpha (HIF-1α) [48]. The latter is commonly overexpressed in many cancers and has many functions such as, increasing glucose metabolism, alteration of apoptotic pathways, stimulating angiogenesis and tumour growth, and invasion [49]. Studies in animal xenograft models revealed that tumour growth and angiogenesis were repressed by the loss of HIF-1 activity and stimulated by HIF-1α overexpression. Also, cancer biopsies from patients indicated that HIF-1α is overexpressed in several tumours and showed that its level of expression correlated with prognosis and mortality [49]. Based on these findings, the activation of HIF-1α in CVD constitutes a potential pro-oncogenic element that may lead to subsequent cancer and constitutes a common therapeutic target for both diseases. However, the presented evidence provides better support to HIF-1α increasing tumour growth rather than causing cancer. Therefore, adequate study design and pre-clinical models are needed to determine whether the HIF-1α initiates cancer.

### Cardiokines stimulate tumour growth

A recent experimental study indicated that factors secreted by failing hearts stimulate tumour growth by releasing pro-oncogenic factors into the circulation [50] (Fig. 6). In the setting of MI, mice genetically predisposed to the development of intestinal tumours, exhibited a higher number of intestinal polyps compared to sham mice. The authors suggested that the oncogenic activity of the failing heart was mediated by secreted factors such as SerpinA3, a factor regulating tumour cell survival pathways and apoptosis [50]. Treating colon cancer cell lines (HT-29) with a selection of proteins that were overexpressed in the myocardium of failing hearts, resulted in a higher proliferative rate. Further, the investigators showed that HF and inflammation biomarkers were associated with new onset cancer incidence among participants with HF from the PREVEND (Prevention of Renal and Vascular End-Stage Disease) study [50]. Comparable outcomes were validated in a recent study showing that MI-induced HF accelerates breast cancer progression in mice. Further, prospective studies showed that cancer prevalence is higher in patients with CV events [51]. Cardiokines may represent a new biological pathway by which CVD induces cancer development and progression. However, these findings require further investigation in larger clinical studies.

### Hypercoagulability promotes cancer

It is already acknowledged that cancer induces a hypercoagulable state through the production of pro-coagulant factors. However, emerging data indicate that thrombosis can also promote cancer (Fig. 6). Higher cancer prevalence was reported in patients after stroke and venous thromboembolism [42, 52]. Moreover, shorter duration of anti-coagulation with warfarin (6 weeks) treatment was associated with a higher risk of incident cancer compared with long-duration treatment (6 months) [53]. Studies indicated that thrombin promotes metastasis and induces vascular growth factors and angiogenesis [54, 55]. Also, thrombin boosts the proliferation of dormant cancer cells and promotes progression to clinical disease [48].

## Cardio-oncology syndrome type IV- (COS 4; indirect): the association between CVD treatments and diagnostics and cancer diagnosis

### Cardiovascular diagnostic radiation

Studies have suggested some indication of an association of cumulative cardiovascular diagnostic radiation with subsequent development of cancer [56–58]. Cancer prevalence attributed to the exposure to cardiovascular diagnostic radiation has been extensively investigated and existing data suggest that children and adolescents are more prone to developing radiation-associated malignancies than older individuals [56–58]. Consequently, there has been a debate regarding the optimal type of imaging modalities that should be used for specific indications in young patients with a focus on reducing radiation-exposing imaging studies.

A retrospective cohort study aimed to evaluate the prevalence of leukaemia and brain tumours after CT scans in children and young adults [56]. The investigators noted a positive link between the radiation dose from CT scans and both leukaemia and brain tumours. However, these cancers are rare, thus, the cumulative absolute risks are small. It has also been estimated that one excess case of leukaemia and one excess case of brain tumour per 10 000 head CT scans will occur within the 10 years after the first CT scan in patients younger than 10 years [56].

Another population-based cohort study compared cancer incidence in young individuals exposed to a CT scan, more than 1 year before cancer diagnosis, with cancer incidence rates in unexposed individuals. Among individuals exposed to a CT scan before the age of 19 years, the overall cancer incidence was 24% higher in comparison to unexposed individuals. The proportional increase in cancer risk was obvious at short intervals of exposure and was greater for patients exposed at younger ages [57]. Further, cancer prevalence in young patients exposed to radiation from cardiac catheterizations is relatively low: below 50 per 100,000 for males and 200 per 100,000 for females [58].

Although clinical benefits should compensate for the low absolute risks, radiation doses should be kept as low as possible and alternative methods, which do not involve ionizing radiation, should be considered if suitable. As an example, ejection fraction can be determined by echocardiography (no ionizing radiation) instead of multigated acquisition scan. Moreover, when considering the annual frequency of CVD requiring examination with CT scans and cardiac catheterization, the overall attributable cancer risk may become important. Therefore, cautious consideration by treating physicians is needed before pursuing any potentially carcinogenic diagnostic or therapeutic options.

### Cardiovascular medications

**Fig. 7:**
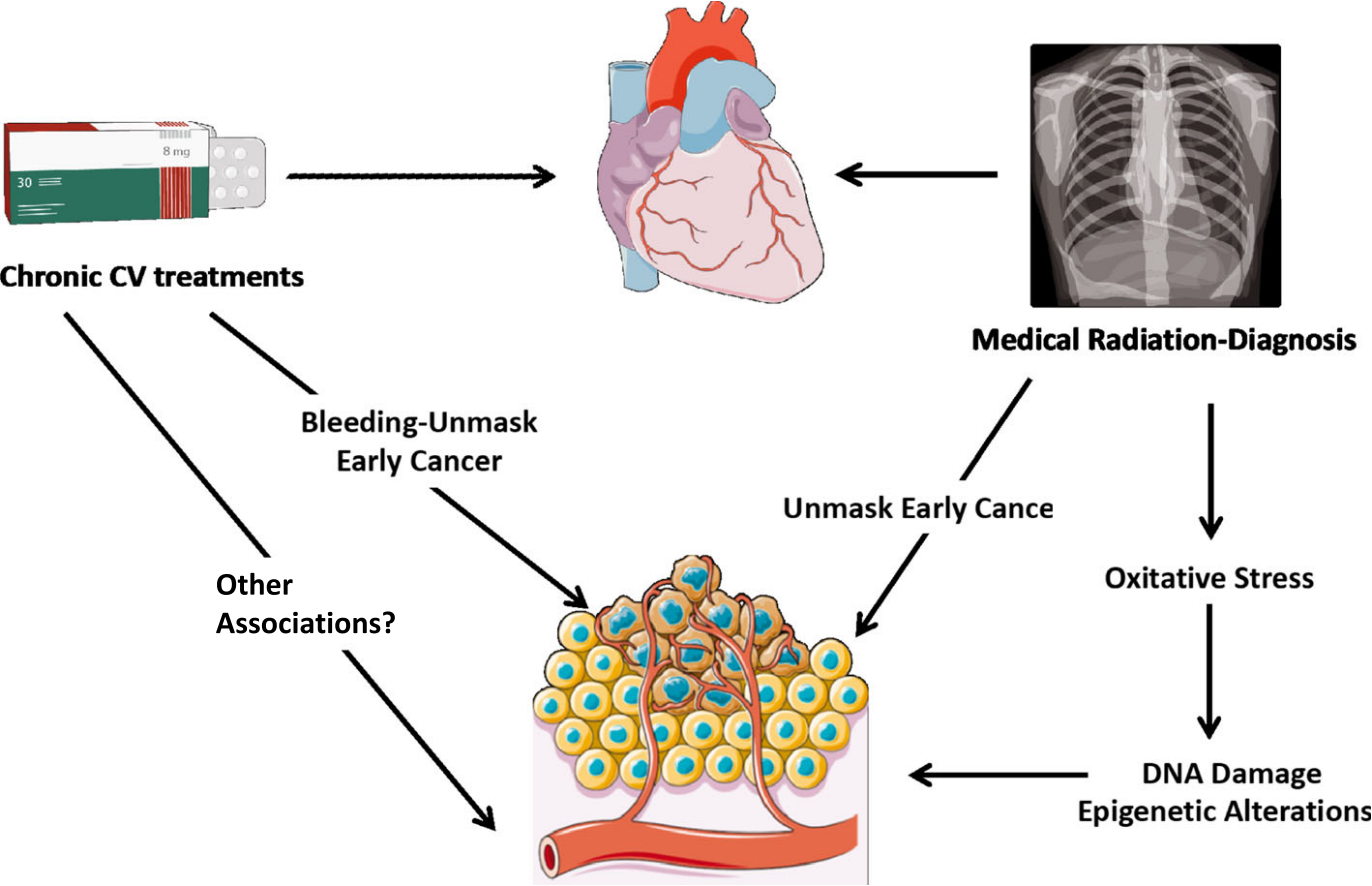
Schema illustrating potential mechanisms in which chronic CVD and/or CVD-associated treatments may associate with cancer diagnosis (Cardio-Oncology Syndrome Type IV). Anticoagulation therapy may result in bleeding which unmasks cancer. Cardiovascular diagnostic radiation, including CT scans and cardiac catheterizations, may result in oxidative stress, DNA damage, and subsequent promotion of a cancer state. CT, Computerized Tomography; CV, cardiovascular

The safety of CVD treatments concerning cancer development is still an active topic of investigation (Fig. 7), with equivocal results. Several studies reported a higher rate of diagnosis of lung cancer in patients treated with angiotensin-converting enzyme inhibitors, especially in individuals treated for more than 5 years [59]. Yet, the outcomes of a large cohort study did not link cancer incidence to angiotensin receptor blocker (ARB) treatment. Interestingly, a subgroup analysis showed a significant association between ARB and cancers in male genital organs [60]. Nevertheless, randomized clinical trials with irbesartan, valsartan, and losartan did not detect any rise in the overall or site-specific cancer risk in patients taking ARB [61]. The inconsistencies among these findings may be due to bias (e.g., selection bias), which can lead to inaccurate conclusions. Evaluating the link between CVD drugs and carcinogenesis is important to consider, given the millions of patients treated with CVD drugs.

### Surveillance bias

Patients with CVD are typically under closer medical surveillance than the general population. Thus, the heightened risk of cancer in CVD might be partially explained by observation bias. Frequent chest X rays and CT scans, as well as PET scans and MRI scans, may unmask pre-existing cancers. Also, repeated lab tests may also raise concern for malignancies, which allows the detection of cancer at early stages. Clinicians have long been aware that bleeding, which is frequently observed in AF patients treated with oral anticoagulants, could potentially unmask occult cancer. Predominantly, evidence of the association between bleeding and cancer diagnosis emerges from small retrospective analyses of databases intended to address other queries. However, a nationwide Danish retrospective study involving patients with AF treated with anticoagulants reported a 15-fold higher hazard of a new colorectal cancer diagnosis with lower gastrointestinal bleeding in patients older than 75 years [62]. A recent study analysed data from the Retrospective observational registry of patients with atrial fibrillation from Vigo’s health area (CardioCHUVIAF). The authors found that gastrointestinal bleeding was associated with a 13-fold higher hazard of a new gastrointestinal cancer diagnosis. In the same study, genitourinary bleeding was associated with an 18-fold higher hazard of new genitourinary cancer diagnosis [63]. A large population of CVD patients is treated with anticoagulants and is at higher bleeding risk. Hence, they are more exposed to clinical management and screening (e.g., colonoscopies), which unmasks occult cancers. To conclude, anticoagulants are likely related to the unmasking of prevalent GI cancers rather than a causative mechanism.

### Survivor bias

An additional source of potential bias is survivor bias for CVD patients that are randomized to a procedure as compared to no procedure. CVD patients that undergo the procedure or receive a study drug may have benefit and live longer, thus leading to increased exposure time at risk for development of subsequent cancer. A classic example of this would be with the use of implantable cardiac defibrillators (ICD) in heart failure with reduced LVEF. Those that receive an ICD may live longer than those without an ICD, leading to increased rates of observed cancer in those with ICDs.

## Cardio-oncology syndrome type V (COS 5): systemic and genetic conditions

### Cardiac tumours

**Fig. 8:**
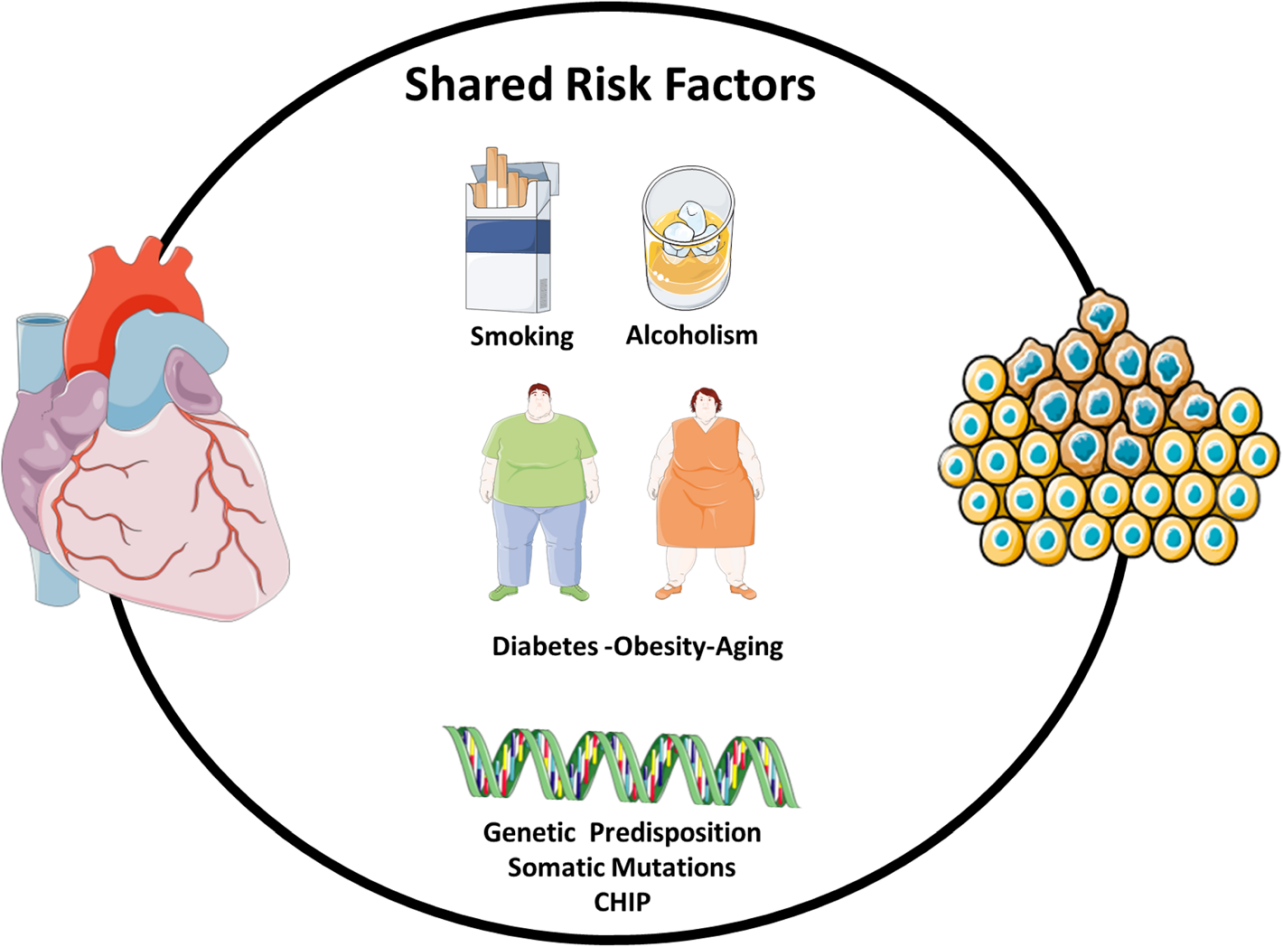
Systemic and genetic conditions may result in concurrent cancer and CVD. Smoking, diabetes, and obesity may increase the risk of both cancer and CVD. Genetic predisposition and somatic mutations may also increase the risk for both disease states. CHiP, clonal haematopoiesis of indeterminate potential; CVD, cardiovascular disease

Cardiac tumours fall within COS Type V (Secondary) due to shared systemic and genetic conditions leading to concurrent cancer and CVD (Fig. 8). The degree of environmental and genetic contribution is largely variable depending on the exact cardiac tumour subtype. Some tumours may occur due to genetic predisposition, but this is not always the case. Perhaps one of the best-known examples of genetically driven cardiac tumours is tuberous sclerosis, an autosomal dominant disorder that is associated with cardiac rhabdomyomas [64]. Another well-known genetic disorder is the Carney complex, which is associated with myxomas [65].

Cardiac tumours can be metastatic and spread via haematogenous seeding (e.g., melanoma, lymphoma), lymphatic spread (e.g., breast cancer), venous extension (e.g., renal cancer), or direct extension (e.g., lung cancer) [66]. Regardless of tumour type, any cardiac mass can have hemodynamic effects depending on the location, by impairing valvular function or obstructing normal blood flow. Additionally, tumours may serve as a nidus for arrhythmias and result in sudden death. Embolic complications can be seen either from the tumour itself or due to thrombus formation on the tumour. More aggressive malignant tumours can also have a direct myocardial or pericardial extension.

### Shared risk factors

Extensive research over the decades has established risk factors associated with the development of cancer and CVD. These factors have been previously interpreted as discrete entities associated with either cancer or CVD. More recently however, an appreciation of a shared overlap model has come to light in regard to risk factors common to both CVD and cancer [67–70].

#### Smoking

Worldwide, smoking accounts for nearly 30% of all cancer-related deaths and increases the risk of CVD 2- to 3-fold. Smoking stimulates pro-inflammatory pathways through irritants, carcinogens, and oxidative stress present in the development of CVD and smoking-related cancer. Impaired nitric oxide bioavailability and endothelial damage caused by smoking are key mediators in the development of atherosclerosis and subsequent CVD [71]. Tobacco is strongly associated with incident lung, oesophageal, pancreatic, and bladder cancer.

#### Alcoholism

Studies over the last several decades have consistently shown a J- or U-shaped relationship between alcohol use and CVD [72]. Compared to non-drinkers, alcohol use increases the risk of colorectal, oropharynx, oesophageal, liver, and breast cancer [73, 74]. Even moderate alcohol use has been associated with an increased risk of overall cancer [75]. Acetaldehyde, an ethanol metabolite, is considered carcinogenic through reactive oxidative stress, altered DNA methylation, and changes in oestrogen pathways [76].

#### Type 2 diabetes Mellitus

Type 2 diabetes mellitus has long been identified as a risk factor for the development of CVD through deleterious effects on the micro- and microvasculature [77]. A consensus report released by the American Diabetic Association found convincing evidence of an association between type 2 diabetes mellitus and cancer [78]. Medications used in the treatment of diabetes also provide insight into the interplay between CVD and cancer. The use of metformin has been reported in several systematic reviews and meta-analyses to reduce the risk of cancer incidence and all-cancer mortality compared to other traditional regimens [79]. More recently, sodium-dependent glucose transporter 2 (SGLT2) inhibitors have become first-line agents in metformin-resistant diabetes and are recommended for secondary CVD prevention due to their cardioprotective effects. In a recent study by Claudio et al., SGLT2 inhibition in early-stage lung adenocarcinoma reduced tumour progression and prolonged survival in mice with SGLT2 expression [80]. Conversely, pioglitazone - a thiazolidinedione - improves glycaemic control in patients with type 2 diabetes but has been shown to have detrimental CV effects and an increased risk of bladder cancer [81].

#### Obesity

This association between CVD and obesity is likely mediated indirectly through factors related to the metabolic syndrome including dyslipidaemia, insulin resistance, sedentary lifestyle, but also a pro-thrombotic and inflammatory state [82, 83]. In a dose-response meta-analysis of observational studies, every 5% increase in body mass index (BMI) was associated with a 10% greater risk of death related to cancer. The risk of cancer associated with obesity is higher in women compared to men and may be attributable to excess adipose tissue producing elevated oestrogen levels, a known risk factor for breast and endometrial cancer [84].

#### Physical inactivity

A dose-response relationship was observed in a pooled meta-analysis, finding 2.5 h/week of moderate-intensity activity led to a significant 13% reduction in cancer mortality [85]. Sedentary behaviour, irrespective of activity level, has also been shown by several meta-analyses to be associated with CVD and certain types of cancer [86]. The shared biological mechanism in reducing CVD and cancer is likely multifactorial through beneficial effects on blood pressure, insulin sensitivity, and reduced adipose tissue [87].

### Shared pathophysiology

While mechanisms involved in the development of CVD in cancer patients have been extensively elucidated, pathways driving the increased prevalence of malignancies in CVD patients have not been equally explored [88]. In this section, we highlight common molecular pathways central to CVD and cancer, namely inflammation and clonal haematopoiesis of indeterminate potential (Fig. 8).

#### Inflammation

Based on the outcomes of the Canakinumab Anti-Inflammatory Thrombosis Outcome Study (CANTOS), inflammation is a valuable target in both cancer and CVD. In this study, patients with MI and elevated CRP were randomly assigned to receive the canakinumab, an IL-1β-targeting antibody. The authors reported lower rates of CV events in patients treated canakinumab compared to the placebo group. Alongside, canakinumab reduced incident lung cancer and cancer-related mortality. These results demonstrate a promising effect of anti-inflammatory agents, such as canakinumab, in reducing CV and cancer events . Accumulating findings from clinical and preclinical studies highlight the role of inflammation in CVD development and consequential complications and highlight the pathogenic activity of proinflammatory cytokines, mainly TNF (tumour necrosis factor)-α, IL (interleukin)-1β, IL-6, and IL-18 [61]. In the context of cancer, targeting cytokines into the tumour by the fusion of anti-cytokine antibodies represents a novel tool in immunotherapy [89]. The inflammatory response to haematological malignancies is often associated with an overproduction of IL-6, predominantly in B cell-derived and plasma cell tumours. Elevated levels of IL-6 promote myocardial injury and are involved in coronary artery diseases [90, 91]. In general, IL-6 elevation has associated with cardiovascular complications such as atherosclerosis, MI, and heart failure. The pathophysiology of ACS and atherosclerosis comprises adaptive immune response T cells and cytokines [92]. Extensive investigations are needed to tease out what factors are key players in the cancer-CVD axis. However, such elements may 1 day serve as therapeutic targets to improve the outcomes in patients.

#### Clonal haematopoiesis of indeterminate potential

Age-related genetic mutations in the highly proliferative hematopoietic stem cells of the bone marrow can lead to clonal haematopoiesis of indeterminate potential Early blood cell progenitors contribute to the creation of a genetically distinct subpopulation of blood cells, and successive mutations in the same clone or subpopulation allow the progression to acute myeloid leukaemia [93]. These mutations occur predominantly in genes that encode for key epigenetic regulators of haematopoiesis, such as Ten-Eleven Translocation-2 (TET2), DNA Methyltransferase 3A (DNMT3α), Additional Sex Combs Like 1 (ASXL1), Janus Kinase 2 (JAK2), and tumour protein 53 (TP53) [94, 95].

Some of these somatic mutations in the hematopoietic stem cells (e.g., TET2, JAK2, and ASXL1) are linked to an increased risk of coronary heart disease [95]. Investigators have reported higher expression of pro-inflammatory cytokines in TET2-deficient macrophages, which stimulates the development of the atherosclerotic plaque. In the same study, the authors observed larger atherosclerotic lesions in mice engrafted with bone marrows of TET2-deficient (homozygous and heterozygous) mice compared to a group of mice engrafted with normal bone marrows. Additional studies have linked dysfunctional TET2 to the stimulation of the cytoplasmic supramolecular assembly Nod-like receptor protein 3 inflammasome (NLRP3) [96]. Additionally, somatic mutations in TET2 and DNMT3α were shown to be associated with a poor prognosis in HF patients [97]. Interestingly, the pharmacological inhibition of 3 (NLRP3) prevented the aggravation of cardiac dysfunction in HF mouse models (TET2 or DNMT3αdeficient mouse models) [98]. Based on the data, genetic risk factors and somatic mutations are emerging as common drivers of cancer and CVD. This holds great clinical relevance as these somatic mutations demonstrate predictive values for cancer and CVD. This promising research may help pave the way for personalized medicine that allows optimized prevention, treatments, and management of patients. 

### Genetic predisposition – monogenetic disease

In this section, we provide instances of common established genetic mutations that may be involved in both diseases. BRCA1/2 mutation carriers are predisposed to breast and ovarian cancer. After the exclusion of deaths due to cancer, the carriers of BRCA1/2 mutations still have lower longevity [99]. Because BRCA1/2 mediates DNA-repair and other vital pathways, it has been previously hypothesized that these genetic variants could be linked to other diseases. Indeed, several studies reported higher levels of proteins or biomarkers associated with elevated thrombotic risk in patients with BRCA1/2 mutations compared to controls with no mutations, independent of breast cancer [100, 101]. These results suggest that the carriers of the mutations are prone to have coagulation-related problems independently of malignancies. Further, low levels of insulin-like growth factor 1 (IGF-1) were detected in individuals carrying BRCA1/2 mutations, while high levels of IGF-1 were detected in BRCA1/2 mutation carriers with breast cancer; abnormal concentrations of IGF-1 have been associated with higher risks to develop insulin resistance, which contributes to the pathophysiology of many CVDs [100, 102].

Titin (TTN) is the largest sarcomeric protein found in the heart and is involved in the pathophysiology of cardiomyopathy. Truncation mutations of TTN are the most frequent mutation observed in dilated cardiomyopathy where most cases are hereditary [103]. TTN mutations, resulting in truncations in the A-band region of the protein, were detected in 25% of patients with dilated cardiomyopathy [104]. Other forms of cardiomyopathies, such as hypertrophic cardiomyopathy and arrhythmogenic right ventricular cardiomyopathy are also linked to TTN mutations [105]. Strikingly, in a cohort that included patients with 34 solid tumours and 7 independent validation cohorts from clinical trials, TTN mutations were detected in ~ 30% of solid tumours. Based on these outcomes, the investigators considered these mutations as potential tools to risk-stratify tumour mutational burden into groups with different clinical responses to immune checkpoint inhibitor monotherapy [106]. These two examples – of what most physicians will consider ‘clear cut monogenetic cancer or CVD examples’ - further underscore the potential links that exist between cancer and CVD.

## Shared prognostic factors

Prognostic markers are hard to objectify because of their either low sensitivity or specificity, unknown thresholds, or difficult reproducibility. Seeing the large overlap in CVD and cancer-related risk factors, it is challenging to find a valuable prognostic biomarker that accurately predicts a patient’s prognosis. Several prognostic biomarkers like age and circulatory biomarkers have extensively been studied in both patients with cancer and CVD [107, 108]. However, due to the low sensitivity and specificity, these biomarkers have yet to be utilized in routine clinical practice. Sex-bias presents as another interesting factor in both CVD and cancer [109, 110]. Gender influences not only the overall incidence and shared risk factors, but also the timing and phenotype of disease presentation, the treatment regimen, and the disease prognosis as well [111].

CV biomarkers have been used in patients with cancer to predict the risk and prognosis of cardiotoxicity, showing promising results [112]. The reverse rationale has also been suggested, reporting that certain tumour biomarkers could predict CVD severity and prognosis. CA 125 known as a biomarker for ovarian cancer and several haematological malignancies has been shown as a promising CV biomarker in diverse CVD [113, 114] . Furthermore, other tumour biomarkers, such as CYFRA 21-1, CEA and Ca 19–9, demonstrated a prognostic value, especially in patients with severe HF [115].

The search for the perfect prognostic biomarker in Cardio-Oncology requires extensive resources and a large data pool. Prognostic biomarkers, both positive and negative ones, could help recognize not only the patients at high CV or cancer risks, but also patients with low CV risk that could benefit from more aggressive treatment regimes.

## Benefits of the proposed classification

We anticipate that the classification system will guide clinicians to better understanding of the predisposing factors and assist with focusing treatment on removing or tempering the driving cause for each disease state. Additionally, these categories may serve as a tool for classifying cardio-oncology patients into relatively more homogenous cohorts for future trials with targeted interventions. Such an approach would help address an existing issue with the field — heterogeneity of cardio-oncology patients and disease states. By focusing on a single classification at a time, this would align treatments with more consistent underlying drivers of disease and provide more accurate estimates of treatment effect.

By understanding the predisposing factor, treatment may be targeted at removing or tempering the driving cause for the disease state. For example, in those with progressive scarring and remodelling of the heart causing a pro-oncogenic environment (Type III), treatment should be targeted at mitigating this effect. Thus, recognizing that heart failure and myocardial infarction can potentially foster a pro-oncogenic environment may lead to intervention studies in patients with heart failure or myocardial infarction specifically targeting components of the pro-oncogenic environment itself, beyond standard treatment for the predisposing cardiovascular diseases. Conversely, if a pro-oncologic environment was caused by CV disease associated treatments or diagnostic imaging (Type IV), the focus would be on weighing and addressing the risks and benefits of considering alternative treatment or imaging modalities, recognizing that these associations remain under investigation. The classification schematic may therefore help researchers identify focused pathways in appropriately selected and relatively homogenous cohorts for future interventions. Lastly, this novel classification system would harmonize understanding and provide a clinical framework for cardiotoxicity from cancer treatment by providing structured definitions with a “universal language” for cardiologists and oncologists when caring for patients.

The identification and management of CV risk factors in all five types of COS are important because if not recognized and treated, both CVD and cancer pose great burdens for our patients. Yet, in those with cancer, the development of CVD poses a greater risk than cancer itself, and those with heart disease have a greater risk of developing cancer as well. Thus, to reduce the risk of adverse outcomes, the traditional CV risk factors should be closely managed in this high-risk population. For instance, lowering cholesterol levels via lifestyle changes, medication, or exercise is believed to diminish breast cancer prevalence and slow-down tumour growth [116, 117]. This can be explained by the fact that cholesterol metabolites stimulate oestrogen receptors, which results in tumour growth [118]. Expanding the role of primary care physicians, oncologists, cardiologists, and allied healthcare providers in cancer survivorship will be essential in identifying and managing these five types of COS in a coordinated fashion.

## Limitations of the proposed classification

We realize that the enormous number of cancer therapies cannot be easily fit into one syndrome or algorithm. Also, aetiologies of both cancer and CV disease are usually multifactorial, so we recognize that our proposal simplifies a complex truth. Collectively, our approach offers a reasonable and practical first attempt to better categorize these patients. We envision our proposed system to be operationalized in a similar manner to the classifications for cardiorenal syndrome [119] or the WHO groups for pulmonary hypertension [120], in which causes and treatment differ by classification group.

The evidence supporting the statement that CVD represents a pro-oncogenic state is based on retrospective and observational outcomes that cannot establish cause and effect. However, three pre-clinical studies with several combinations of HF aetiologies and cancer types have proven the direct connection between the two syndromes. Also, investigators of these studies found that failing hearts can affect tumour growth and severity independent of CVD risk factors [50, 121, 122].

**Fig. 9:**
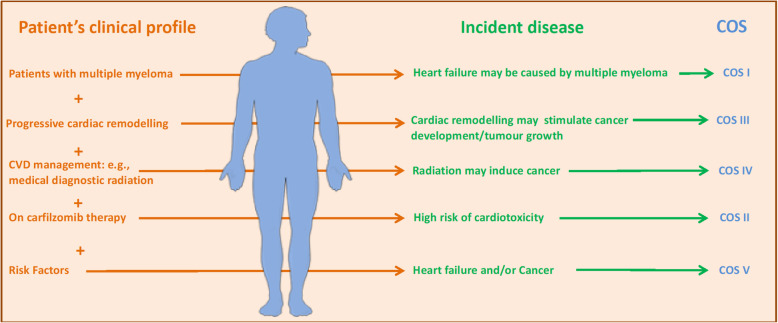
Theoretical depiction of a clinical situation with a patient consistent with more than a single classification. However, the application of our classification system depends on which disease is diagnosed first and on the available data regarding systemic and genetic predisposition and risk factors [122, 123]

In the clinical practice, we acknowledge the difficulty in discerning between the CVD itself and the underlying risk factors. Thus, it is more challenging to determine whether detecting incident cancer following CVD is based on shared risk factors such as a history of smoking (Type 5), or if there is truly a link between CVD and cancer (Type 3). Nevertheless, the classification system is essential as it allows clinicians to manage patients and evaluate the predisposition to one or both of the diseases. Different strategies or treatments may be more effective within each defined category. Even in instances where a patient may share more than a single classification (Fig. 9), recognition of which specific categories apply will still guide treatment strategies. Future studies should validate the usefulness of our proposal, and diagnostic and therapeutic studies will be necessary to prove clinical benefit.

## Conclusion

**Fig. 10:**
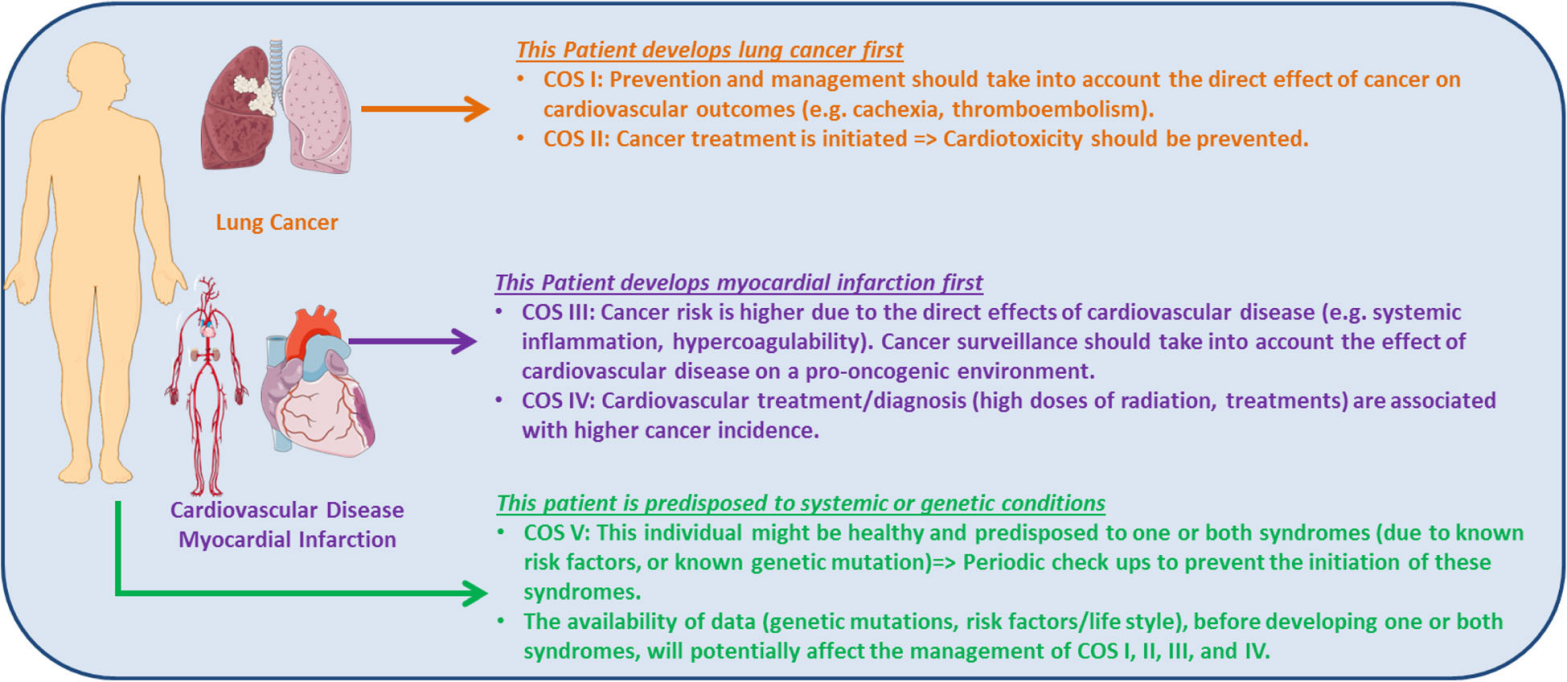
Theoretical depiction of how the classification can be useful in the clinical setting

In this review, we propose a classification system to categorize Cardio-Oncology syndromes into five distinct groups. The classification system (I-V) can help separate and unmask certain aetiologies of CV damage from cancer and cancer treatment, and vice versa (Fig. 9). Using the classification system, the course of treatment selected by oncologists (COS I, COS II, COS V) or cardiologists (COS III, COS IV, COS V) may help guide surveillance and management pursued by the converse specialty (Fig. 10). This classification has emerged in response to observations of the effects of cancer and its therapies on the CV system, as well as the development or diagnosis of cancers in the setting of heart disease. These aetiologies include the effects of the tumour itself on the CV system which can have a direct effect on CV dysfunction (COS Type I) from aetiologies including TLS, cardiac cachexia, and arterial thromboembolism; the indirect effects from anti-neoplastic treatments (COS Type II) encompassing traditional chemotherapy, targeted therapies, and immunotherapy, as well as radiation therapy; CVD pro-oncogenic effect (COS Type III), which exerts direct effects from cardiokines, galectin-3 and other factors; chronic CVD and their associated treatments (COS Type IV), which may increase the risk for or enhanced detection of cancer; and lastly shared risk factors, metabolic diseases, and genetic predisposition (COS Type V), which work synergistically to increase the risk of long-term cardiac complications, as well as malignancies. This classification method paves the way for improvements in patient care, research, and education in Cardio-Oncology.. Having such a framework facilitates providing the best cancer care while optimizing CV health, and also being able to pre-empt both CVD and new malignancies based on the recognition of underlying or pre-existing risk and disease.

## Data Availability

Not applicable.

## Abbreviations

COS: Cardio-oncology syndromes
CVD: Cardiovascular disease
TLS: Tumour lysis syndrome
MI: Myocardial infarction
HF: Heart failure
HER2: Human epidermal growth factor receptor 2
AF: Atrial fibrillation
mTORC: Mammalian target of rapamycin complex
CAR-T: Chimeric antigen receptor T
CRS: Cytokine release syndrome
ACS: Acute coronary syndrome
RE-LY: Randomized evaluation long-term anticoagulant therapy study
WHS: Women’s Health Study
HIF-1α: Hypoxia-inducible factor 1-alpha
PREVEND: Prevention of renal and vascular end-stage disease
ALLHAT: Lipid-lowering treatment to prevent heart attack trial
ACEIs: Angiotensin-converting enzyme inhibitor
CHARM: Candesartan HF assessment of reduction in mortality and morbidity
SGLT2: Sodium-dependent glucose transporter 2
BMI: Body mass index
CANTOS: Canakinumab anti-inflammatory thrombosis outcome study
CAR: Chimeric antigen receptor
TET2: Ten-eleven translocation-2
DNMT3α: DNA Methyltransferase 3A
ASXL1: Additional Sex Combs Like 1
JAK2: Janus Kinase 2
TP53: Tumour protein 53
NLRP3: Nod-like receptor protein 3 inflammasome
IGF-1: Insulin-like growth factor 1

## References

[1] Survivorship NCIOoC. Statistics: National Cancer Institute: Statistics: National Cancer Institute 2019 2019. Available from: https://cancercontrol.cancer.gov/ocs/statistics/index.html. Accessed 2020.

[2] Siegel RL, Miller KD, Jemal A (2020). Cancer statistics, 2020. CA Cancer J Clin.

[3] Aboumsallem JP, Moslehi J, de Boer RA (2020). Reverse cardio-oncology: cancer development in patients with cardiovascular disease. J Am Heart Assoc..

[4] Bhatia N, Santos M, Jones LW, Beckman JA, Penson DF, Morgans AK (2016). Cardiovascular effects of androgen deprivation therapy for the treatment of prostate Cancer: ABCDE steps to reduce cardiovascular disease in patients with prostate Cancer. Circulation..

[5] Mehta LS, Watson KE, Barac A, Beckie TM, Bittner V, Cruz-Flores S, Dent S, Kondapalli L, Ky B, Okwuosa T, Piña IL, Volgman AS, American Heart Association Cardiovascular Disease in Women and Special Populations Committee of the Council on Clinical Cardiology; Council on Cardiovascular and Stroke Nursing; and Council on Quality of Care and Outcomes Research (2018). Cardiovascular disease and breast Cancer: where these entities intersect: a scientific statement from the American Heart Association. Circulation..

[6] Moslehi J, Zhang Q, Moore KJ (2020). Crosstalk between the heart and Cancer: beyond drug toxicity. Circulation..

[7] Banke A, Schou M, Videbaek L, Moller JE, Torp-Pedersen C, Gustafsson F (2016). Incidence of cancer in patients with chronic heart failure: a long-term follow-up study. Eur J Heart Fail.

[8] Hasin T, Gerber Y, McNallan SM, Weston SA, Kushwaha SS, Nelson TJ (2013). Patients with heart failure have an increased risk of incident cancer. J Am Coll Cardiol.

[9] Hasin T, Gerber Y, Weston SA, Jiang R, Killian JM, Manemann SM, Cerhan JR, Roger VL (2016). Heart failure after myocardial infarction is associated with increased risk of Cancer. J Am Coll Cardiol.

[10] Navi BB, Reiner AS, Kamel H, Iadecola C, Okin PM, Elkind MS (2017). Risk of arterial thromboembolism in patients with cancer. J Am Coll Cardiol.

[11] Brenner B, Bikdeli B, Tzoran I, Madridano O, López-Reyes R, Suriñach JM, Blanco-Molina Á, Tufano A, Núñez JJL, Trujillo-Santos J, Monreal M, RIETE Investigators (2018). Arterial ischemic events are a major complication in cancer patients with venous thromboembolism. Am J Med.

[12] Mukai M, Oka T (2018). Mechanism and management of cancer-associated thrombosis. J Cardiol.

[13] Hisada Y, Mackman N (2017). Cancer-associated pathways and biomarkers of venous thrombosis. Blood..

[14] Baracos VE, Martin L, Korc M, Guttridge DC, Fearon KCH (2018). Cancer-associated cachexia. Nat Rev Dis Primers.

[15] Belloum Y, Rannou-Bekono F, Favier FB (2017). Cancer-induced cardiac cachexia: pathogenesis and impact of physical activity (review). Oncol Rep.

[16] Cosper PF, Leinwand LA. Cancer causes cardiac atrophy and autophagy in a sexually dimorphic Manne*r. cancer* Res 2011;71(5):1710–20, doi: 10.1158/0008-5472.CAN-10-3145. 10.1158/0008-5472.CAN-10-3145 PMC3049989 21163868

[17] Ezeoke CC, Morley JE (2015). Pathophysiology of anorexia in the cancer cachexia syndrome. J Cachexia Sarcopenia Muscle.

[18] Sardar MR, Greway A, DeAngelis M, Tysko EO, Lehmann S, Wohlstetter M, Patel R (2015). Cardiovascular impact of eating disorders in adults: a single center experience and literature review. Heart Views.

[19] Mirrakhimov AE, Voore P, Khan M, Ali AM (2015). Tumor lysis syndrome: a clinical review. World J Crit Care Med.

[20] Chaudhary K, Malhotra K, Sowers J, Aroor A (2013). Uric acid - key ingredient in the recipe for cardiorenal metabolic syndrome. Cardiorenal Med.

[21] Bracun V, Aboumsallem JP, van der Meer P, de Boer RA (2020). Cardiac biomarkers in patients with Cancer: considerations, clinical implications, and future avenues. Curr Oncol Rep.

[22] Perez IE, Taveras Alam S, Hernandez GA, Sancassani R. Cancer therapy-related cardiac dysfunction: an overview for the clinician. Clin Med Insights Cardiol. 2019;13:1179546819866445. 10.1177/1179546819866445 PMC6664629 31384135

[23] Brown SA. Preventive cardio-oncology: the time has come. Front Cardiovasc Med. 2019;6:187. 10.3389/fcvm.2019.00187 PMC6965025 31998754

[24] Chang HM, Okwuosa TM, Scarabelli T, Moudgil R, Yeh ETH (2017). Cardiovascular complications of Cancer therapy: Best practices in diagnosis, prevention, and management: part 2. J Am Coll Cardiol.

[25] Herrmann J, Yang EH, Iliescu CA, Cilingiroglu M, Charitakis K, Hakeem A, Toutouzas K, Leesar MA, Grines CL, Marmagkiolis K (2016). Vascular toxicities of Cancer therapies: the old and the new--an evolving avenue. Circulation..

[26] Brown SA, Nhola L, Herrmann J (2017). Cardiovascular toxicities of small molecule tyrosine kinase inhibitors: an opportunity for systems-based approaches. Clin Pharmacol Ther.

[27] Brown SA, Ray JC, Herrmann J. Precision cardio-oncology: a systems-based perspective on cardiotoxicity of tyrosine kinase inhibitors and immune checkpoint inhibitors. J Cardiovasc Transl Res. 2020. https://pubmed.ncbi.nlm.nih.gov/32253744/. 10.1007/s12265-020-09992-5 PMC8855704 32253744

[28] Chaar M, Kamta J, Ait-Oudhia S (2018). Mechanisms, monitoring, and management of tyrosine kinase inhibitors-associated cardiovascular toxicities. Onco Targets Ther.

[29] Giavridis T, van der Stegen SJC, Eyquem J, Hamieh M, Piersigilli A, Sadelain M (2018). CAR T cell-induced cytokine release syndrome is mediated by macrophages and abated by IL-1 blockade. Nat Med.

[30] Shimabukuro-Vornhagen A, Gödel P, Subklewe M, Stemmler HJ, Schlößer HA, Schlaak M, Kochanek M, Böll B, von Bergwelt-Baildon MS (2018). Cytokine release syndrome. J Immunother Cancer.

[31] Davila ML, Riviere I, Wang X, Bartido S, Park J, Curran K, et al. Efficacy and toxicity management of 19-28z CAR T cell therapy in B cell acute lymphoblastic leukemia. Sci Transl Med. 2014;6(224):224ra25-ra25. 10.1126/scitranslmed.3008226 PMC4684949 24553386

[32] Maude SL, Frey N, Shaw PA, Aplenc R, Barrett DM, Bunin NJ, Chew A, Gonzalez VE, Zheng Z, Lacey SF, Mahnke YD, Melenhorst JJ, Rheingold SR, Shen A, Teachey DT, Levine BL, June CH, Porter DL, Grupp SA (2014). Chimeric antigen receptor T cells for sustained remissions in leukemia. N Engl J Med.

[33] Le RQ, Li L, Yuan W, Shord SS, Nie L, Habtemariam BA (2018). FDA approval summary: tocilizumab for treatment of chimeric antigen receptor T cell-induced severe or life-threatening cytokine release syndrome. Oncologist..

[34] Abboud R, Keller J, Slade M, DiPersio JF, Westervelt P, Rettig MP (2016). Severe cytokine-release syndrome after T cell-replete peripheral blood Haploidentical donor transplantation is associated with poor survival and anti-IL-6 therapy is safe and well tolerated. Biol Blood Marrow Transplant.

[35] Jamal FA, Khaled SK (2020). The cardiovascular complications of chimeric antigen receptor T cell therapy. Curr Hematol Malig Rep.

[36] Alvi RM, Frigault MJ, Fradley MG, Jain MD, Mahmood SS, Awadalla M (2019). Cardiovascular events among adults treated with chimeric antigen receptor T-cells (CAR-T). J Am Coll Cardiol.

[37] Hernandez C, Huebener P, Schwabe RF (2016). Damage-associated molecular patterns in cancer: a double-edged sword. Oncogene..

[38] Maude SL, Barrett D, Teachey DT, Grupp SA (2014). Managing cytokine release syndrome associated with novel T cell-engaging therapies. Cancer J.

[39] Mahmood SS, Fradley MG, Cohen JV, Nohria A, Reynolds KL, Heinzerling LM, Sullivan RJ, Damrongwatanasuk R, Chen CL, Gupta D, Kirchberger MC, Awadalla M, Hassan MZO, Moslehi JJ, Shah SP, Ganatra S, Thavendiranathan P, Lawrence DP, Groarke JD, Neilan TG (2018). Myocarditis in patients treated with immune checkpoint inhibitors. J Am Coll Cardiol.

[40] Malmborg M, Christiansen CB, Schmiegelow MD, Torp-Pedersen C, Gislason G, Schou M (2018). Incidence of new onset cancer in patients with a myocardial infarction - a nationwide cohort study. BMC Cardiovasc Disord.

[41] Berton G, Cordiano R, Cavuto F, Bagato F, Segafredo B, Pasquinucci M (2018). Neoplastic disease after acute coronary syndrome: incidence, duration, and features: the ABC-4* study on heart disease. J Cardiovasc Med (Hagerstown).

[42] Baron JA, Gridley G, Weiderpass E, Nyren O, Linet M (1998). Venous thromboembolism and cancer. Lancet..

[43] Qureshi AI, Malik AA, Saeed O, Adil MM, Rodriguez GJ, Suri MF (2015). Incident cancer in a cohort of 3,247 cancer diagnosis free ischemic stroke patients. Cerebrovasc Dis.

[44] Seretis A, Cividini S, Markozannes G, Tseretopoulou X, Lopez DS, Ntzani EE, Tsilidis KK (2019). Association between blood pressure and risk of cancer development: a systematic review and meta-analysis of observational studies. Sci Rep.

[45] Han H, Guo W, Shi W, Yu Y, Zhang Y, Ye X, He J (2017). Hypertension and breast cancer risk: a systematic review and meta-analysis. Sci Rep.

[46] Marijon E, Le Heuzey JY, Connolly S, Yang S, Pogue J, Brueckmann M (2013). Causes of death and influencing factors in patients with atrial fibrillation: a competing-risk analysis from the randomized evaluation of long-term anticoagulant therapy study. Circulation..

[47] Conen D, Wong JA, Sandhu RK, Cook NR, Lee IM, Buring JE (2016). Risk of malignant Cancer among women with new-onset atrial fibrillation. JAMA Cardiol.

[48] Hasin T, Iakobishvili Z, Weisz G (2017). Associated risk of malignancy in patients with cardiovascular disease: evidence and possible mechanism. Am J Med.

[49] Semenza GL (2002). Involvement of hypoxia-inducible factor 1 in human cancer. Intern Med.

[50] Meijers WC, Maglione M, Bakker SJL, Oberhuber R, Kieneker LM, de Jong S, Haubner BJ, Nagengast WB, Lyon AR, van der Vegt B, van Veldhuisen DJ, Westenbrink BD, van der Meer P, Silljé HHW, de Boer RA (2018). Heart failure stimulates tumor growth by circulating factors. Circulation..

[51] Koelwyn GJ, Newman AAC, Afonso MS, van Solingen C, Corr EM, Brown EJ, et al. Myocardial infarction accelerates breast cancer via innate immune reprogramming. Nat Med. 2020. https://pubmed.ncbi.nlm.nih.gov/32661390/. 10.1038/s41591-020-0964-7 PMC7789095 32661390

[52] Sorensen HT, Mellemkjaer L, Steffensen FH, Olsen JH, Nielsen GL (1998). The risk of a diagnosis of cancer after primary deep venous thrombosis or pulmonary embolism. N Engl J Med.

[53] Schulman S, Lindmarker P (2000). Incidence of cancer after prophylaxis with warfarin against recurrent venous thromboembolism. Duration of anticoagulation trial. N Engl J Med.

[54] Haralabopoulos GC, Grant DS, Kleinman HK, Maragoudakis ME (1997). Thrombin promotes endothelial cell alignment in Matrigel in vitro and angiogenesis in vivo. Am J Phys.

[55] Caunt M, Huang YQ, Brooks PC, Karpatkin S (2003). Thrombin induces neoangiogenesis in the chick chorioallantoic membrane. J Thromb Haemost.

[56] Pearce MS, Salotti JA, Little MP, McHugh K, Lee C, Kim KP, Howe NL, Ronckers CM, Rajaraman P, Craft AW, Parker L, Berrington de González A (2012). Radiation exposure from CT scans in childhood and subsequent risk of leukaemia and brain tumours: a retrospective cohort study. Lancet..

[57] Mathews JD, Forsythe AV, Brady Z, Butler MW, Goergen SK, Byrnes GB, Giles GG, Wallace AB, Anderson PR, Guiver TA, McGale P, Cain TM, Dowty JG, Bickerstaffe AC, Darby SC (2013). Cancer risk in 680,000 people exposed to computed tomography scans in childhood or adolescence: data linkage study of 11 million Australians. BMJ..

[58] Harbron RW, Chapple CL, O'Sullivan JJ, Best KE (2017). Berrington de Gonzalez a, Pearce MS. survival adjusted cancer risks attributable to radiation exposure from cardiac catheterisations in children. Heart..

[59] Hicks BM, Filion KB, Yin H, Sakr L, Udell JA, Azoulay L (2018). Angiotensin converting enzyme inhibitors and risk of lung cancer: population based cohort study. BMJ..

[60] Pasternak B, Svanstrom H, Callreus T, Melbye M, Hviid A (2011). Use of angiotensin receptor blockers and the risk of cancer. Circulation..

[61] Collaboration ARBT (2011). Effects of telmisartan, irbesartan, valsartan, candesartan, and losartan on cancers in 15 trials enrolling 138,769 individuals. J Hypertens.

[62] Rasmussen PV, Dalgaard F, Gislason GH, Brandes A, Johnsen SP, Grove EL, et al. Gastrointestinal bleeding and the risk of colorectal cancer in anticoagulated patients with atrial fibrillation. Eur Heart J. 2020. https://pubmed.ncbi.nlm.nih.gov/32030399/. 10.1093/eurheartj/ehz964 32030399

[63] Raposeiras Roubin S, Abu Assi E, Barreiro Pardal C, Cespon Fernandez M, Munoz Pousa I, Cobas Paz R (2020). New Cancer diagnosis after bleeding in anticoagulated patients with atrial fibrillation. J Am Heart Assoc.

[64] Hinton RB, Prakash A, Romp RL, Krueger DA, Knilans TK (2014). International Tuberous Sclerosis Consensus G. Cardiovascular manifestations of tuberous sclerosis complex and summary of the revised diagnostic criteria and surveillance and management recommendations from the International Tuberous Sclerosis Consensus Group. J Am Heart Assoc.

[65] Casey M, Vaughan CJ, He J, Hatcher CJ, Winter JM, Weremowicz S, Montgomery K, Kucherlapati R, Morton CC, Basson CT (2000). Mutations in the protein kinase a R1alpha regulatory subunit cause familial cardiac myxomas and carney complex. J Clin Invest.

[66] Maleszewski JJ, Bois MC, Bois JP, Young PM, Stulak JM, Klarich KW (2018). Neoplasia and the heart: pathological review of effects with clinical and radiological correlation. J Am Coll Cardiol.

[67] de Boer RA, Meijers WC, van der Meer P, van Veldhuisen DJ (2019). Cancer and heart disease: associations and relations. Eur J Heart Fail.

[68] Meijers WC, de Boer RA (2019). Common risk factors for heart failure and cancer. Cardiovasc Res.

[69] Koene RJ, Prizment AE, Blaes A, Konety SH (2016). Shared risk factors in cardiovascular disease and cancer. Circulation..

[70] Forouzanfar MH, Afshin A, Alexander LT, Anderson HR, Bhutta ZA, Biryukov S, Brauer M, Burnett R, Cercy K, Charlson FJ, Cohen AJ, Dandona L, Estep K, Ferrari AJ, Frostad JJ, Fullman N, Gething PW, Godwin WW, Griswold M, Hay SI, Kinfu Y, Kyu HH, Larson HJ, Liang X, Lim SS, Liu PY, Lopez AD, Lozano R, Marczak L, Mensah GA, Mokdad AH, Moradi-Lakeh M, Naghavi M, Neal B, Reitsma MB, Roth GA, Salomon JA, Sur PJ, Vos T, Wagner JA, Wang H, Zhao Y, Zhou M, Aasvang GM, Abajobir AA, Abate KH, Abbafati C, Abbas KM, Abd-Allah F, Abdulle AM, Abera SF, Abraham B, Abu-Raddad LJ, Abyu GY, Adebiyi AO, Adedeji IA, Ademi Z, Adou AK, Adsuar JC, Agardh EE, Agarwal A, Agrawal A, Kiadaliri AA, Ajala ON, Akinyemiju TF, al-Aly Z, Alam K, Alam NKM, Aldhahri SF, Aldridge RW, Alemu ZA, Ali R, Alkerwi A', Alla F, Allebeck P, Alsharif U, Altirkawi KA, Martin EA, Alvis-Guzman N, Amare AT, Amberbir A, Amegah AK, Amini H, Ammar W, Amrock SM, Andersen HH, Anderson BO, Antonio CAT, Anwari P, Ärnlöv J, Artaman A, Asayesh H, Asghar RJ, Assadi R, Atique S, Avokpaho EFGA, Awasthi A, Quintanilla BPA, Azzopardi P, Bacha U, Badawi A, Bahit MC, Balakrishnan K, Barac A, Barber RM, Barker-Collo SL, Bärnighausen T, Barquera S, Barregard L, Barrero LH, Basu S, Batis C, Bazargan-Hejazi S, Beardsley J, Bedi N, Beghi E, Bell B, Bell ML, Bello AK, Bennett DA, Bensenor IM, Berhane A, Bernabé E, Betsu BD, Beyene AS, Bhala N, Bhansali A, Bhatt S, Biadgilign S, Bikbov B, Bisanzio D, Bjertness E, Blore JD, Borschmann R, Boufous S, Bourne RRA, Brainin M, Brazinova A, Breitborde NJK, Brenner H, Broday DM, Brugha TS, Brunekreef B, Butt ZA, Cahill LE, Calabria B, Campos-Nonato IR, Cárdenas R, Carpenter DO, Carrero JJ, Casey DC, Castañeda-Orjuela CA, Rivas JC, Castro RE, Catalá-López F, Chang JC, Chiang PPC, Chibalabala M, Chimed-Ochir O, Chisumpa VH, Chitheer AA, Choi JYJ, Christensen H, Christopher DJ, Ciobanu LG, Coates MM, Colquhoun SM, Manzano AGC, Cooper LT, Cooperrider K, Cornaby L, Cortinovis M, Crump JA, Cuevas-Nasu L, Damasceno A, Dandona R, Darby SC, Dargan PI, das Neves J, Davis AC, Davletov K, de Castro EF, de la Cruz-Góngora V, de Leo D, Degenhardt L, del Gobbo LC, del Pozo-Cruz B, Dellavalle RP, Deribew A, Jarlais DCD, Dharmaratne SD, Dhillon PK, Diaz-Torné C, Dicker D, Ding EL, Dorsey ER, Doyle KE, Driscoll TR, Duan L, Dubey M, Duncan BB, Elyazar I, Endries AY, Ermakov SP, Erskine HE, Eshrati B, Esteghamati A, Fahimi S, Faraon EJA, Farid TA, Farinha CSS, Faro A, Farvid MS, Farzadfar F, Feigin VL, Fereshtehnejad SM, Fernandes JG, Fischer F, Fitchett JRA, Fleming T, Foigt N, Foreman K, Fowkes FGR, Franklin RC, Fürst T, Futran ND, Gakidou E, Garcia-Basteiro AL, Gebrehiwot TT, Gebremedhin AT, Geleijnse JM, Gessner BD, Giref AZ, Giroud M, Gishu MD, Giussani G, Goenka S, Gomez-Cabrera MC, Gomez-Dantes H, Gona P, Goodridge A, Gopalani SV, Gotay CC, Goto A, Gouda HN, Gugnani HC, Guillemin F, Guo Y, Gupta R, Gupta R, Gutiérrez RA, Haagsma JA, Hafezi-Nejad N, Haile D, Hailu GB, Halasa YA, Hamadeh RR, Hamidi S, Handal AJ, Hankey GJ, Hao Y, Harb HL, Harikrishnan S, Haro JM, Hassanvand MS, Hassen TA, Havmoeller R, Heredia-Pi IB, Hernández-Llanes NF, Heydarpour P, Hoek HW, Hoffman HJ, Horino M, Horita N, Hosgood HD, Hoy DG, Hsairi M, Htet AS, Hu G, Huang JJ, Husseini A, Hutchings SJ, Huybrechts I, Iburg KM, Idrisov BT, Ileanu BV, Inoue M, Jacobs TA, Jacobsen KH, Jahanmehr N, Jakovljevic MB, Jansen HAFM, Jassal SK, Javanbakht M, Jayaraman SP, Jayatilleke AU, Jee SH, Jeemon P, Jha V, Jiang Y, Jibat T, Jin Y, Johnson CO, Jonas JB, Kabir Z, Kalkonde Y, Kamal R, Kan H, Karch A, Karema CK, Karimkhani C, Kasaeian A, Kaul A, Kawakami N, Kazi DS, Keiyoro PN, Kemmer L, Kemp AH, Kengne AP, Keren A, Kesavachandran CN, Khader YS, Khan AR, Khan EA, Khan G, Khang YH, Khatibzadeh S, Khera S, Khoja TAM, Khubchandani J, Kieling C, Kim CI, Kim D, Kimokoti RW, Kissoon N, Kivipelto M, Knibbs LD, Kokubo Y, Kopec JA, Koul PA, Koyanagi A, Kravchenko M, Kromhout H, Krueger H, Ku T, Defo BK, Kuchenbecker RS, Bicer BK, Kuipers EJ, Kumar GA, Kwan GF, Lal DK, Lalloo R, Lallukka T, Lan Q, Larsson A, Latif AA, Lawrynowicz AEB, Leasher JL, Leigh J, Leung J, Levi M, Li X, Li Y, Liang J, Liu S, Lloyd BK, Logroscino G, Lotufo PA, Lunevicius R, MacIntyre M, Mahdavi M, Majdan M, Majeed A, Malekzadeh R, Malta DC, Manamo WAA, Mapoma CC, Marcenes W, Martin RV, Martinez-Raga J, Masiye F, Matsushita K, Matzopoulos R, Mayosi BM, McGrath JJ, McKee M, Meaney PA, Medina C, Mehari A, Mejia-Rodriguez F, Mekonnen AB, Melaku YA, Memish ZA, Mendoza W, Mensink GBM, Meretoja A, Meretoja TJ, Mesfin YM, Mhimbira FA, Millear A, Miller TR, Mills EJ, Mirarefin M, Misganaw A, Mock CN, Mohammadi A, Mohammed S, Mola GLD, Monasta L, Hernandez JCM, Montico M, Morawska L, Mori R, Mozaffarian D, Mueller UO, Mullany E, Mumford JE, Murthy GVS, Nachega JB, Naheed A, Nangia V, Nassiri N, Newton JN, Ng M, Nguyen QL, Nisar MI, Pete PMN, Norheim OF, Norman RE, Norrving B, Nyakarahuka L, Obermeyer CM, Ogbo FA, Oh IH, Oladimeji O, Olivares PR, Olsen H, Olusanya BO, Olusanya JO, Opio JN, Oren E, Orozco R, Ortiz A, Ota E, PA M, Pana A, Park EK, Parry CD, Parsaeian M, Patel T, Caicedo AJP, Patil ST, Patten SB, Patton GC, Pearce N, Pereira DM, Perico N, Pesudovs K, Petzold M, Phillips MR, Piel FB, Pillay JD, Plass D, Polinder S, Pond CD, Pope CA, Pope D, Popova S, Poulton RG, Pourmalek F, Prasad NM, Qorbani M, Rabiee RHS, Radfar A, Rafay A, Rahimi-Movaghar V, Rahman M, Rahman MHU, Rahman SU, Rai RK, Rajsic S, Raju M, Ram U, Rana SM, Ranganathan K, Rao P, García CAR, Refaat AH, Rehm CD, Rehm J, Reinig N, Remuzzi G, Resnikoff S, Ribeiro AL, Rivera JA, Roba HS, Rodriguez A, Rodriguez-Ramirez S, Rojas-Rueda D, Roman Y, Ronfani L, Roshandel G, Rothenbacher D, Roy A, Saleh MM, Sanabria JR, Sanchez-Riera L, Sanchez-Niño MD, Sánchez-Pimienta TG, Sandar L, Santomauro DF, Santos IS, Sarmiento-Suarez R, Sartorius B, Satpathy M, Savic M, Sawhney M, Schmidhuber J, Schmidt MI, Schneider IJC, Schöttker B, Schutte AE, Schwebel DC, Scott JG, Seedat S, Sepanlou SG, Servan-Mori EE, Shaddick G, Shaheen A, Shahraz S, Shaikh MA, Levy TS, Sharma R, She J, Sheikhbahaei S, Shen J, Sheth KN, Shi P, Shibuya K, Shigematsu M, Shin MJ, Shiri R, Shishani K, Shiue I, Shrime MG, Sigfusdottir ID, Silva DAS, Silveira DGA, Silverberg JI, Simard EP, Sindi S, Singh A, Singh JA, Singh PK, Slepak EL, Soljak M, Soneji S, Sorensen RJD, Sposato LA, Sreeramareddy CT, Stathopoulou V, Steckling N, Steel N, Stein DJ, Stein MB, Stöckl H, Stranges S, Stroumpoulis K, Sunguya BF, Swaminathan S, Sykes BL, Szoeke CEI, Tabarés-Seisdedos R, Takahashi K, Talongwa RT, Tandon N, Tanne D, Tavakkoli M, Taye BW, Taylor HR, Tedla BA, Tefera WM, Tegegne TK, Tekle DY, Terkawi AS, Thakur JS, Thomas BA, Thomas ML, Thomson AJ, Thorne-Lyman AL, Thrift AG, Thurston GD, Tillmann T, Tobe-Gai R, Tobollik M, Topor-Madry R, Topouzis F, Towbin JA, Tran BX, Dimbuene ZT, Tsilimparis N, Tura AK, Tuzcu EM, Tyrovolas S, Ukwaja KN, Undurraga EA, Uneke CJ, Uthman OA, van Donkelaar A, van Os J, Varakin YY, Vasankari T, Veerman JL, Venketasubramanian N, Violante FS, Vollset SE, Wagner GR, Waller SG, Wang JL, Wang L, Wang Y, Weichenthal S, Weiderpass E, Weintraub RG, Werdecker A, Westerman R, Whiteford HA, Wijeratne T, Wiysonge CS, Wolfe CDA, Won S, Woolf AD, Wubshet M, Xavier D, Xu G, Yadav AK, Yakob B, Yalew AZ, Yano Y, Yaseri M, Ye P, Yip P, Yonemoto N, Yoon SJ, Younis MZ, Yu C, Zaidi Z, Zaki MES, Zhu J, Zipkin B, Zodpey S, Zuhlke LJ, Murray CJL (2016). Global, regional, and national comparative risk assessment of 79 behavioural, environmental and occupational, and metabolic risks or clusters of risks, 1990–2015: a systematic analysis for the global burden of disease study 2015. Lancet.

[71] Morris PB, Ference BA, Jahangir E, Feldman DN, Ryan JJ, Bahrami H, el-Chami MF, Bhakta S, Winchester DE, al-Mallah MH, Sanchez Shields M, Deedwania P, Mehta LS, Phan BAP, Benowitz NL (2015). Cardiovascular effects of exposure to cigarette smoke and electronic cigarettes: clinical perspectives from the prevention of cardiovascular disease section leadership council and early career councils of the American College of Cardiology. J Am Coll Cardiol.

[72] Di Castelnuovo A, Costanzo S, Bagnardi V, Donati MB, Iacoviello L, De Gaetano G (2006). Alcohol dosing and total mortality in men and women: an updated meta-analysis of 34 prospective studies. Arch Intern Med.

[73] Baan R, Straif K, Grosse Y, Secretan B, El Ghissassi F, Bouvard V (2007). Carcinogenicity of alcoholic beverages. Lancet Oncol.

[74] Bagnardi V, Rota M, Botteri E, Tramacere I, Islami F, Fedirko V, Scotti L, Jenab M, Turati F, Pasquali E, Pelucchi C, Galeone C, Bellocco R, Negri E, Corrao G, Boffetta P, la Vecchia C (2015). Alcohol consumption and site-specific cancer risk: a comprehensive dose–response meta-analysis. Br J Cancer.

[75] Cao Y, Willett WC, Rimm EB, Stampfer MJ, Giovannucci EL (2015). Light to moderate intake of alcohol, drinking patterns, and risk of cancer: results from two prospective US cohort studies. BMJ..

[76] Seitz HK, Stickel F (2007). Molecular mechanisms of alcohol-mediated carcinogenesis. Nat Rev Cancer.

[77] Mellitus D (1999). A major risk factor for cardiovascular disease: a joint editorial statement by the American Diabetes Association; the National Heart, Lung, and Blood Institute; the juvenile Diabetes Foundation international; the National Institute of Diabetes and Digestive and Kidney Diseases; and the American Heart Association. Circulation..

[78] Giovannucci E, Harlan DM, Archer MC, Bergenstal RM, Gapstur SM, Habel LA, Pollak M, Regensteiner JG, Yee D (2010). Diabetes and cancer: a consensus report. CA Cancer J Clin.

[79] Noto H, Goto A, Tsujimoto T, Noda M (2012). Cancer risk in diabetic patients treated with metformin: a systematic review and meta-analysis. Plos One.

[80] Scafoglio CR, Villegas B, Abdelhady G, Bailey ST, Liu J, Shirali AS (2018). Sodium-glucose transporter 2 is a diagnostic and therapeutic target for early-stage lung adenocarcinoma. Sci Transl Med.

[81] Lipscombe LL, Gomes T, Lévesque LE, Hux JE, Juurlink DN, Alter DA (2007). Thiazolidinediones and cardiovascular outcomes in older patients with diabetes. JAMA..

[82] Aboumsallem JP, Muthuramu I, Mishra M, Kempen H, De Geest B. Effective Treatment of Diabetic Cardiomyopathy and Heart Failure with Reconstituted HDL (Milano) in Mice. Int J Mol Sci. 2019;20(6). https://pubmed.ncbi.nlm.nih.gov/30871282/. 10.3390/ijms20061273 PMC6470758 30871282

[83] Aboumsallem JP, Muthuramu I, Mishra M, De Geest B. Cholesterol-Lowering Gene Therapy Prevents Heart Failure with Preserved Ejection Fraction in Obese Type 2 Diabetic Mice. Int J Mol Sci. 2019;20(9). https://www.ncbi.nlm.nih.gov/pmc/articles/PMC6539537/. 10.3390/ijms20092222 PMC6539537 31064116

[84] Calle EE, Rodriguez C, Walker-Thurmond K, Thun MJ (2003). Overweight, obesity, and mortality from cancer in a prospectively studied cohort of US adults. N Engl J Med.

[85] Li T, Wei S, Shi Y, Pang S, Qin Q, Yin J, Deng Y, Chen Q, Wei S, Nie S, Liu L (2016). The dose–response effect of physical activity on cancer mortality: findings from 71 prospective cohort studies. Br J Sports Med.

[86] Biswas A, Oh PI, Faulkner GE, Bajaj RR, Silver MA, Mitchell MS, Alter DA (2015). Sedentary time and its association with risk for disease incidence, mortality, and hospitalization in adults: a systematic review and meta-analysis. Ann Intern Med.

[87] McTiernan A (2008). Mechanisms linking physical activity with cancer. Nat Rev Cancer.

[88] de Boer RA, Hulot J-S, Tocchetti CG, Aboumsallem JP, ..., et al. Common mechanistic pathways in cancer and heart failure. A scientific roadmap on behalf of the Translational Research Committee of the Heart Failure Association (HFA) of the European Society of Cardiology (ESC). Eur J Heart Fail. 2020; 22(12):2272–89. 10.1002/ejhf.2029. 10.1002/ejhf.2029 PMC7894564 33094495

[89] Lode HN, Reisfeld RA (2000). Targeted cytokines for cancer immunotherapy. Immunol Res.

[90] Kanda T, Takahashi T (2004). Interleukin-6 and cardiovascular diseases. Jpn Heart J.

[91] Burger R (2013). Impact of interleukin-6 in hematological malignancies. Transfus Med Hemother.

[92] Libby P, Hansson GK (2019). From focal lipid storage to systemic inflammation: JACC review topic of the week. J Am Coll Cardiol.

[93] Libby P, Sidlow R, Lin AE, Gupta D, Jones LW, Moslehi J, Zeiher A, Jaiswal S, Schulz C, Blankstein R, Bolton KL, Steensma D, Levine RL, Ebert BL (2019). Clonal hematopoiesis: crossroads of aging, cardiovascular disease, and Cancer: JACC review topic of the week. J Am Coll Cardiol.

[94] Acuna-Hidalgo R, Sengul H, Steehouwer M, van de Vorst M, Vermeulen SH, Kiemeney L (2017). Ultra-sensitive sequencing identifies high prevalence of clonal hematopoiesis-associated mutations throughout adult life. Am J Hum Genet.

[95] Jaiswal S, Natarajan P, Silver AJ, Gibson CJ, Bick AG, Shvartz E, McConkey M, Gupta N, Gabriel S, Ardissino D, Baber U, Mehran R, Fuster V, Danesh J, Frossard P, Saleheen D, Melander O, Sukhova GK, Neuberg D, Libby P, Kathiresan S, Ebert BL (2017). Clonal hematopoiesis and risk of atherosclerotic cardiovascular disease. N Engl J Med.

[96] Libby P (2017). Interleukin-1 Beta as a target for atherosclerosis therapy: biological basis of CANTOS and beyond. J Am Coll Cardiol.

[97] Dorsheimer L, Assmus B, Rasper T, Ortmann CA, Ecke A, Abou-El-Ardat K (2019). Association of Mutations Contributing to clonal hematopoiesis with prognosis in chronic ischemic heart failure. JAMA Cardiol.

[98] Cremer S, Schloss MJ, Vinegoni C, Foy BH, Zhang S, Rohde D, Hulsmans M, Fumene Feruglio P, Schmidt S, Wojtkiewicz G, Higgins JM, Weissleder R, Swirski FK, Nahrendorf M (2020). Diminished reactive hematopoiesis and cardiac inflammation in a mouse model of recurrent myocardial infarction. J Am Coll Cardiol.

[99] Mai PL, Chatterjee N, Hartge P, Tucker M, Brody L, Struewing JP (2009). Potential excess mortality in BRCA1/2 mutation carriers beyond breast, ovarian, prostate, and pancreatic cancers, and melanoma. Plos One.

[100] Perez-Segura P, Zamorano-Leon JJ, Acosta D, Santos-Sancho JM, Modrego J, Caldes T (2016). BRCA2 gene mutations and coagulation-associated biomarkers. Thromb Haemost.

[101] Custodio A, Lopez-Farre AJ, Zamorano-Leon JJ, Mateos-Caceres PJ, Macaya C, Caldes T (2012). Changes in the expression of plasma proteins associated with thrombosis in BRCA1 mutation carriers. J Cancer Res Clin Oncol.

[102] van Westerop LL, Arts-de Jong M, Hoogerbrugge N, de Hullu JA, Maas AH (2016). Cardiovascular risk of BRCA1/2 mutation carriers: a review. Maturitas..

[103] LeWinter MM, Granzier HL (2013). Titin is a major human disease gene. Circulation..

[104] Granzier HL, Irving TC (1995). Passive tension in cardiac muscle: contribution of collagen, titin, microtubules, and intermediate filaments. Biophys J.

[105] Brun F, Barnes CV, Sinagra G, Slavov D, Barbati G, Zhu X, Graw SL, Spezzacatene A, Pinamonti B, Merlo M, Salcedo EE, Sauer WH, Taylor MR, Mestroni L, Familial Cardiomyopathy Registry (2014). Titin and desmosomal genes in the natural history of arrhythmogenic right ventricular cardiomyopathy. J Med Genet.

[106] Jia Q, Wang J, He N, He J, Zhu B. Titin mutation associated with responsiveness to checkpoint blockades in solid tumors. JCI Insight. 2019;4(10). https://pubmed.ncbi.nlm.nih.gov/31092729/. 10.1172/jci.insight.127901 PMC6542599 31092729

[107] Nicoll R, Wiklund U, Zhao Y, Diederichsen A, Mickley H, Ovrehus K (2016). Gender and age effects on risk factor-based prediction of coronary artery calcium in symptomatic patients: a euro-CCAD study. Atherosclerosis..

[108] Ye J, Luo QY, Wang XP, Liu ZY, Chen MX, Huang H, Zhang L (2019). Serum apolipoprotein A-I combined with C-reactive protein serves as a novel prognostic stratification system for colorectal Cance*r*. Cancer Manag Res.

[109] Pelliccia F, Gaudio C (2018). The elusive link between sex hormone levels and Takotsubo syndrome. Int J Cardiol.

[110] Udell JA, Koh M, Qiu F, Austin PC, Wijeysundera HC, Bagai A, et al. Outcomes of Women and Men With Acute Coronary Syndrome Treated With and Without Percutaneous Coronary Revascularization. J Am Heart Assoc. 2017;6(1). https://www.ncbi.nlm.nih.gov/pmc/articles/PMC5523628/. 10.1161/JAHA.116.004319 PMC5523628 28108465

[111] Lam CSP, Arnott C, Beale AL, Chandramouli C, Hilfiker-Kleiner D, Kaye DM (2019). Sex differences in heart failure. Eur Heart J.

[112] Bracun V, de Boer RA (2020). Troponins and natriuretic peptides to detect cardiotoxicity: useful biomarkers or paradise lost?. Eur J Heart Fail.

[113] Nunez J, Minana G, Nunez E, Chorro FJ, Bodi V, Sanchis J (2014). Clinical utility of antigen carbohydrate 125 in heart failure. Heart Fail Rev.

[114] Soler M, Minana G, Santas E, Nunez E, de la Espriella R, Valero E (2020). CA125 outperforms NT-proBNP in acute heart failure with severe tricuspid regurgitation. Int J Cardiol.

[115] Shi C, van der Wal HH, Sillje HHW, Dokter MM, van den Berg F, Huizinga L (2020). Tumour biomarkers: association with heart failure outcomes. J Intern Med.

[116] Ahern TP, Pedersen L, Tarp M, Cronin-Fenton DP, Garne JP, Silliman RA, Sorensen HT, Lash TL (2011). Statin prescriptions and breast cancer recurrence risk: a Danish nationwide prospective cohort study. J Natl Cancer Inst.

[117] Holmes MD, Chen WY, Feskanich D, Kroenke CH, Colditz GA (2005). Physical activity and survival after breast cancer diagnosis. JAMA..

[118] Nelson ER, Wardell SE, Jasper JS, Park S, Suchindran S, Howe MK (2013). 27-hydroxycholesterol links hypercholesterolemia and breast cancer pathophysiology. Science..

[119] Ronco C, Haapio M, Anavekar N, Bellomo R, House AA (2008). Cardiorenal syndrome. J Am Coll Cardiol.

[120] Simonneau G, Robbins IM, Beghetti M, Channick RN, Delcroix M, Denton CP, Elliott CG, Gaine SP, Gladwin MT, Jing ZC, Krowka MJ, Langleben D, Nakanishi N, Souza R (2009). Updated clinical classification of pulmonary hypertension. J Am Coll Cardiol.

[121] Koelwyn GJ, Newman AAC, Afonso MS, van Solingen C, Corr EM, Brown EJ, Albers KB, Yamaguchi N, Narke D, Schlegel M, Sharma M, Shanley LC, Barrett TJ, Rahman K, Mezzano V, Fisher EA, Park DS, Newman JD, Quail DF, Nelson ER, Caan BJ, Jones LW, Moore KJ (2020). Myocardial infarction accelerates breast cancer via innate immune reprogramming. Nat Med.

[122] Avraham S, Abu-Sharki S, Shofti R, Haas T, Korin B, Kalfon R, Friedman T, Shiran A, Saliba W, Shaked Y, Aronheim A (2020). Early cardiac remodeling promotes tumor growth and metastasis. Circulation..

[123] Waxman AJ, Clasen S, Hwang WT, Garfall A, Vogl DT, Carver J, O’Quinn R, Cohen AD, Stadtmauer EA, Ky B, Weiss BM (2018). Carfilzomib-associated cardiovascular adverse events: a systematic review and meta-analysis. JAMA Oncol.

